# Regulation of STAT3 and its role in cardioprotection by conditioning: focus on non-genomic roles targeting mitochondrial function

**DOI:** 10.1007/s00395-021-00898-0

**Published:** 2021-10-12

**Authors:** Stefano Comità, Saveria Femmino, Cecilia Thairi, Giuseppe Alloatti, Kerstin Boengler, Pasquale Pagliaro, Claudia Penna

**Affiliations:** 1 Department of Clinical and Biological Sciences, University of Turin, Regione Gonzole 10, Orbassano, 10043 Torino, TO Italy; 2 Department of Medical Sciences, University of Turin, Torino, Italy; 3 Uni-Astiss, Polo Universitario Rita Levi Montalcini, Asti, Italy; 4 Institute of Physiology, University of Giessen, Giessen, Germany

**Keywords:** Ca^2+^ regulation, Ischemia reperfusion injury, Mitochondrial permeability transition pore, Reactive oxygen species, RISK pathway, SAFE pathway, STAT3, STAT5

## Abstract

Ischemia–reperfusion injury (IRI) is one of the biggest challenges for cardiovascular researchers given the huge death toll caused by myocardial ischemic disease. Cardioprotective conditioning strategies, namely pre- and post-conditioning maneuvers, represent the most important strategies for stimulating pro-survival pathways essential to preserve cardiac health. Conditioning maneuvers have proved to be fundamental for the knowledge of the molecular basis of both IRI and cardioprotection. Among this evidence, the importance of signal transducer and activator of transcription 3 (STAT3) emerged. STAT3 is not only a transcription factor but also exhibits non-genomic pro-survival functions preserving mitochondrial function from IRI. Indeed, STAT3 is emerging as an influencer of mitochondrial function to explain the cardioprotection phenomena. Studying cardioprotection, STAT3 proved to be crucial as an element of the survivor activating factor enhancement (SAFE) pathway, which converges on mitochondria and influences their function by cross-talking with other cardioprotective pathways. Clearly there are still some functional properties of STAT3 to be discovered. Therefore, in this review, we highlight the evidence that places STAT3 as a promoter of the metabolic network. In particular, we focus on the possible interactions of STAT3 with processes aimed at maintaining mitochondrial functions, including the regulation of the electron transport chain, the production of reactive oxygen species, the homeostasis of Ca^2+^ and the inhibition of opening of mitochondrial permeability transition pore. Then we consider the role of STAT3 and the parallels between STA3/STAT5 in cardioprotection by conditioning, giving emphasis to the human heart and confounders.

## Introduction

Global health is constantly threatened by cardiovascular diseases (CVDs) and every year 31% of worldwide deaths are caused by fatal CVDs. According to the World Health Organization, CVDs caused 17.9 million deaths in 2016. Among these, 85% are attributed to ischemic heart disease or stroke [186].

Over the past twenty to thirty years, mortality from ischemic heart disease has significantly decreased. However, currently available therapies improve symptoms and slow pathological heart remodeling but fail to further reduce CVD mortality [178]. A complete understanding of the molecular targets and mechanisms underlying heart disease is certainly the key to developing new and effective therapeutic strategies.

Coronary reperfusion is currently the “gold standard” for saving the lives of patients with acute myocardial infarction (MI). Reperfusion is mandatory to avoid fatal damage to the ischemic myocardium. However, the reperfusion itself contributes to exacerbating the damage and the extent of the infarct size (IS). In this regard, cardiovascular research over the years has investigated how to limit ischemia–reperfusion injury (IRI), leading to the development of cardioprotective maneuvers, which allowed researchers to analyze and understand the molecular aspects of IRI [77].

Already in 1972 Braunwald and collaborators suggested that the infarct area was not only a function of the duration of ischemia, the area at risk and collateral flow, but could also be reduced with cardioprotective interventions [144]. However, only in 1986 Murry et al. [153] demonstrated in an animal model that a few minutes of coronary occlusion interspersed with a few minutes of reperfusion reduced the area of myocardial infarction caused by a subsequent period of prolonged lethal ischemia/reperfusion (IR). This was the discovery of ischemic preconditioning (IPreC) as a powerful endogenous cardioprotective phenomenon. However, only 4 years later another laboratory confirmed this original observation [130]. Since that time, the IS-limiting effects of IPreC have been demonstrated in all species tested, including humans and have also been shown to be effective in the multicenter network of experimental research centers that made up the Consortium for preclinicAl assESsment of cARdioprotective therapies (CAESAR) [94]. It was then demonstrated that the preconditioned state resulted from protective signal transduction [226]. It was discovered that a short coronary occlusion releases ligands (e.g., adenosine, bradykinin, opioids, platelet activating factor and/or sphingosine) which acting on their receptors trigger protective cascades that converge on protein kinase C (PKC) [138, 166] and on mitochondria [137]. The production of reactive oxygen species (ROS) with a trigger role for cardioprotection also plays an important role in this mechanism [212]. The importance of mitochondria in these phenomena is certain, as much of the cell death in the heart is due to the formation of mitochondrial permeability transition pores (mPTPs) in the first minutes of reperfusion, which is prevented by conditioning procedures [75, 78].

**Fig. 1:**
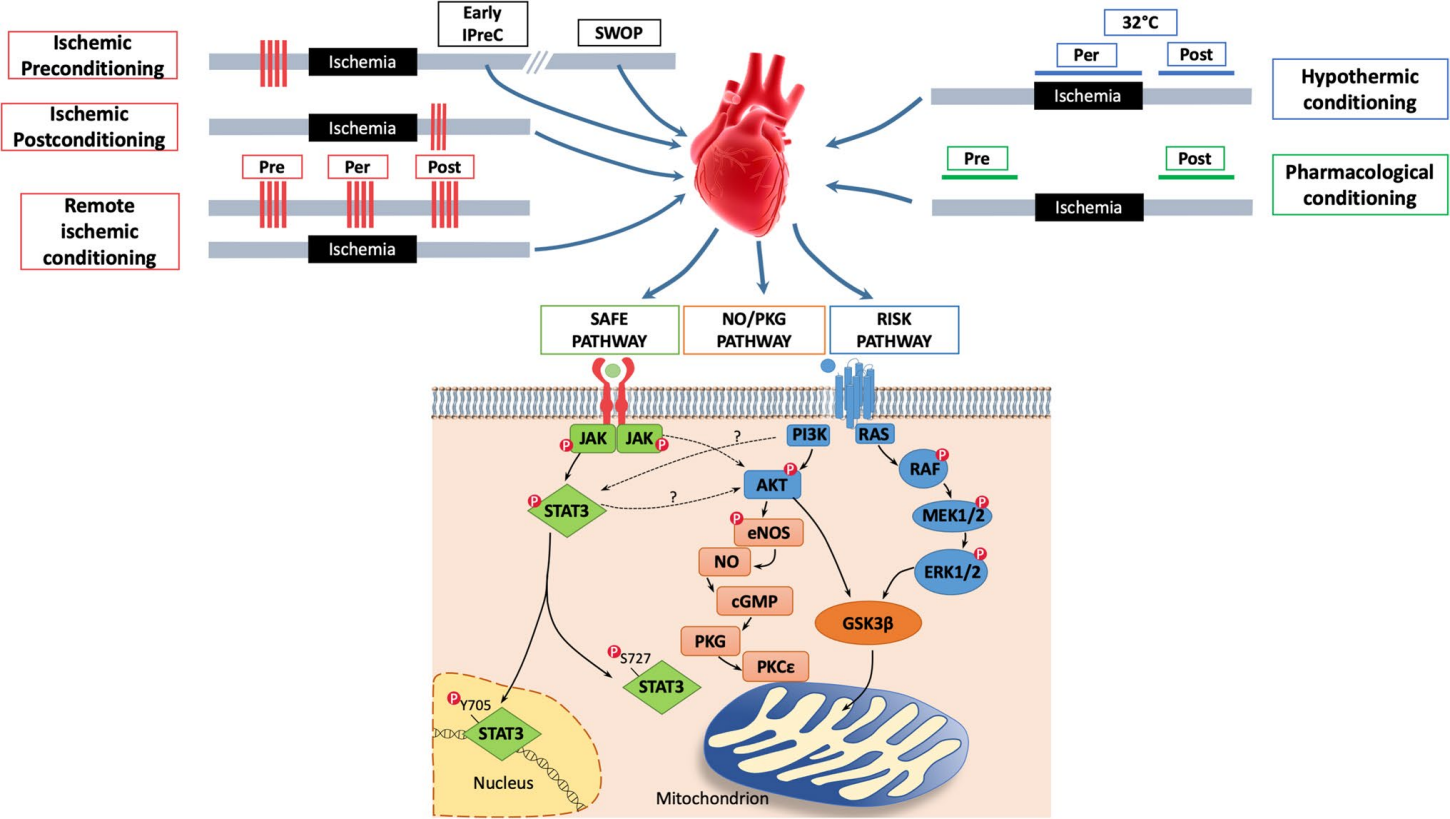
Cardiac conditioning can be induced by brief periods of ischemia (red lines), that is ischemic preconditioning (IPreC), postconditioning (IPostC) and remote conditioning (RIC), as well as by hypothermic or pharmacological interventions. Pre-, Per- and Postconditioning are able to protect the heart *vs* IRI, by triggering three main transduction pathways, SAFE, RISK and NO/PKG pathways; these pathways can cross-talk and result in the activation of anti-apoptotic stimuli and the preservation of mitochondrial function. *Akt* Protein kinase B, *cGMP* cyclic Guanosine Monophosphate, *eNOS* endothelial Nitric Oxide Synthase, *ERK1/2* Extracellular Receptor Kinase ½, *GSK3β* Glycogen Synthase Kinase-3β, *IPreC* Ischemic PreConditioning, *JAK* Janus Kinase, *MAPK* Mitogen-Activated Protein Kinase, *MEK* MAPK/ERK Kinase, *NO* Nitric Oxide, *PI3K* PhosphatidylInositol-4,5-bisphosphate 3-Kinase, *PKCε* Protein Kinase C ε subtype, *PKG* Protein Kinase G, *RAF* serine/threonine kinase, *RAS* rat Sarcoma Virus, *RISK* Reperfusion Injury Salvage Kinase, *SAFE* Survivor Activating Factor Enhancement, *STAT3* Signal Transducer and Activator of Transcription 3

**Fig. 2:**
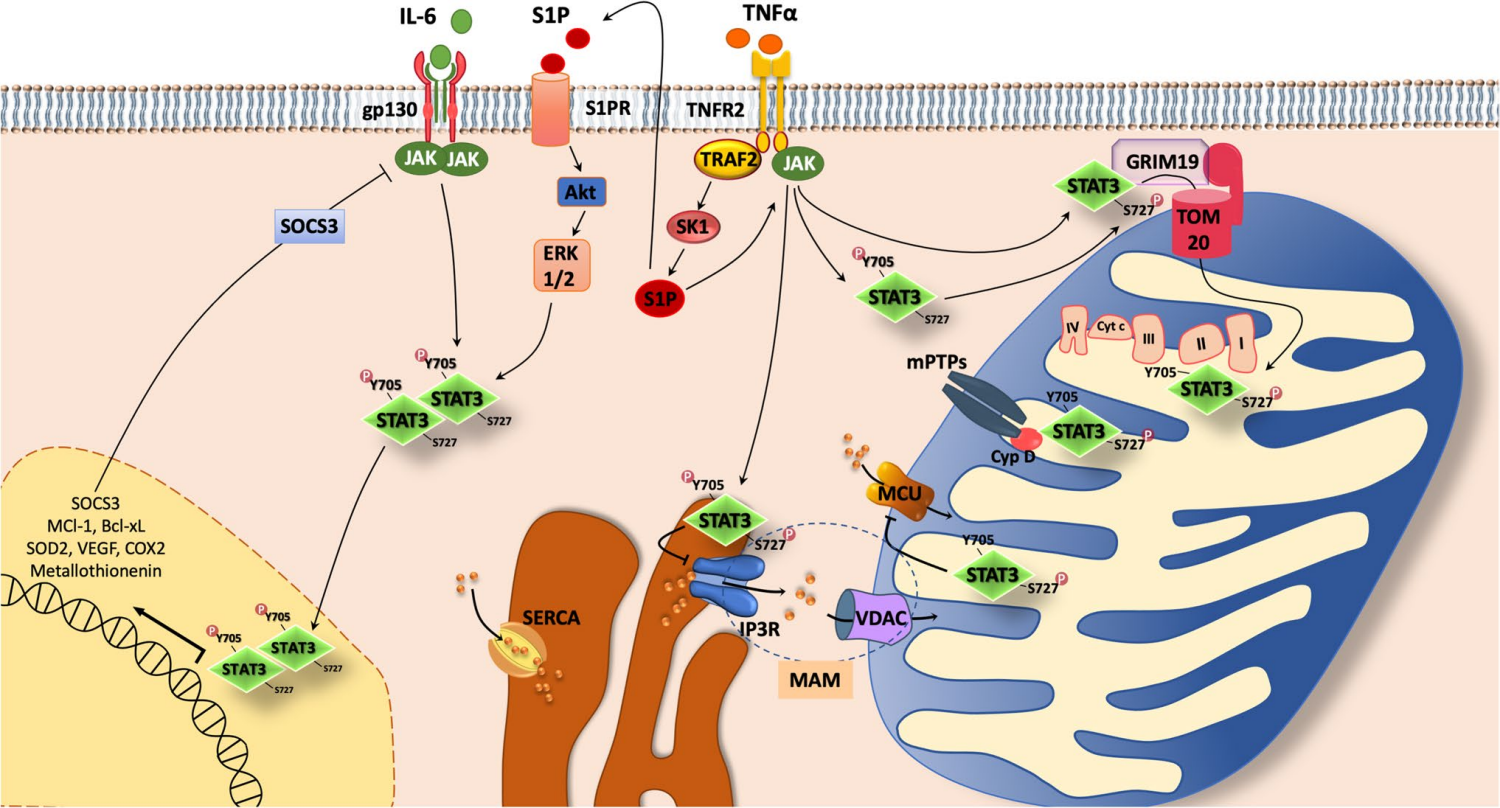
STAT3 canonical and non-canonical pathways. In SAFE pathway JAK/STAT3 can be activated upon activation of receptors including gp130, TNFR2, S1PR. Other molecules (not represented in figure) may activate SAFE pathway, including HDL, Melatonin, Erythropoietin, Insulin and Leptin. Upon phosphorylation at Y705 and dimerization, STAT3 dimer travels to the nucleus, where it regulates gene transcription. The phosphorylation at S727 may allow STAT3 to interact with GRIM19 and TOM20 to enter the mitochondria. STAT3 has been shown to interact with multiple mitochondrial proteins, such as MCU, promoting mitochondrial Ca^2+^ entry, complex I and II of the ETC, increasing ATP level and decreasing ROS production. STAT3 interacting with CypD prevents the opening of mPTPs. STAT3 has been detected also in the MAM fraction, where it is able to promote IPR3 degradation and prevent cytosolic Ca^2+^ accumulation. *Akt* Protein kinase B, *ATP* adenosine triphosphate, *Bcl-xL* B-cell lymphoma-extra large, *COX2* Cytochrome c oxidase subunit 2, *CypD* Cyclophilin D, *Cyt C* Cytochrome C, *ER* Endoplasmic Reticulum, *ERK1/2* Extracellular Receptor Kinase ½, *ETC* Electron Transport Chain, *gp130* glycoprotein 130, *GRIM19* Gene associated with Retinoid Interferon-induced cell Mortality 19, *HDL *High Density Lipoprotein; IP3R, Inositol 1,4,5-trisphosphate Receptors,* JAK *Janus Kinase,* MAM *Mitochondrial-Associated ER Membrane,* MCl-1 *Myeloid cell leukemia 1,* MCU *Mitochondrial Calcium Uniporter,* mPTP *mitochondrial Permeability Transition Pore,* ROS Reactive oxygen species*,* S1P *Sphingosine-1-Phosphate,* S1PR *Sphingosine-1-Phosphate Receptors,* SAFE *Survivor Activating Factor Enhancement,* SERCA *Sarco-Endoplasmic Reticulum Calcium ATPase,* SK1 *Sphingosine Kinase 1,* SOCS3 *Suppressor of Cytokine Signaling 3*; SOD2 *Superoxide dismutase 2*, STAT3 *Signal Transducer and Activator of Transcription 3*, TNFR2 *Tumor Necrosis Factor Receptor 2;* TOM20*Translocase of the outer membrane 20,* TRAF2 *TNF-Receptor-Associated-Factor 2,* VDAC *Voltage-Dependent Anion Channels,* VEGF *Vascular endothelial growth factor

The cardioprotective pathways are not yet fully understood, but in the early 2000s, Yellon's group [72, 182] proposed the reperfusion injury salvage kinase (RISK) pathway, which includes pro-survival kinases that must be activated at the time of reperfusion to protect against IRI (Fig. 1). Then, studies have shown that it is possible to protect the heart with short intermittent phases of ischemia applied at the beginning of reperfusion, ischemic postconditioning (IPostC) [215, 248], as well as by ischemia in tissues and organs remote to the heart, remote ischemic conditioning (RIC) [171]. Indeed, these conditioning maneuvers trigger via multiple mechanisms various cardioprotective pathways besides the RISK, including the NO/PKG (nitric oxide/cyclic guanosine monophosphate (cGMP)-dependent protein kinase G; Fig. 1), and the so-called survivor activating factor enhancement (SAFE) pathways, which can cross-talk (Figs. 1 and 2) [71, 197, 209]. The SAFE pathway was proposed in 2009 by Lecour et al. [113], it considers a central role for the signal transducer and activator of transcription 3 (STAT3) and will be described in more detail throughout this review article.

The importance of the RISK and/or SAFE pathway has been demonstrated in all forms of ischemic conditioning (IPreC, IPostC and RIC) as well as in that obtained with substances administered before or after infarcting ischemia (*pharmacological conditioning*) [69, 238]. The mechanisms induced by pharmacological conditioning are similar to IPreC and IPostC, with the difference that pharmacological conditioning could be applied in a less invasive way. Indeed, in animal and clinical studies, many substances have shown to induce protection against myocardial IR, including volatile anesthetics, opioids, some anti-diabetic drugs, statins but also endogenous substances and hormones, such as adenosine, leptin, insulin and ghrelin [71, 114, 115, 168]. In addition, *hypothermic conditioning* by mild hypothermia (32–35 °C) has been described in the contest of myocardial protection and STAT3 in animal and in clinical studies [88, 161, 163]. It is now clear that targeting intra-cardiomyocytes pathways together a multitarget approach is necessary for a successful cardioprotective strategy (EU-CARDIOPROTECTION COST Action CA16225) [39].

Of note, in preconditioning obtained with interventions/agents, the cardioprotection occurs in two phases, in which STAT3 has been seen to play a pivotal role, as member of the SAFE pathway. The first phase is called classical or early preconditioning and lasts 2–3 h. The second phase is called second window of protection (SWOP) or delayed or late preconditioning. It starts about 12 h after the IPreC maneuvers and lasts 48–72 h [73].

Although, STAT3 is involved as a transcription factor inducing the upregulation of antioxidant, antiapoptotic and pro-angiogenic genes in SWOP [22, 251], it has been recently observed in cardiac mitochondria in the maintenance of electron transport chain (ETC) activity and in the prevention of opening of mPTPs [225, 236]. In addition, the mitochondrial activity of STAT3 is emerging as an important factor and may represent the needle of the balance in mechanisms that confer cardioprotection. Notwithstanding that some studies reported the role of STAT3 in cardiac conditioning, others evidenced the important involvement of STAT5 for the translation of cardioprotection [e.g.*,* 56, 80].

Therefore, in this review we first briefly describe the structure of STAT3 and the principal STAT3 post-translational modifications. Then we focus on the role of STAT3 within mitochondria highlighting the evidence that places STAT3 as a promoter of the metabolic network. In particular, we focus on the possible interactions of STAT3 with the ETC, the production of ROS, the Ca^2+^ homeostasis, the mitochondria quality control and the inhibition of mPTPs opening. We also provide a critical evaluation of pathophysiological roles of STAT3 and the parallels between STA3/STAT5 in the context of cardiac IRI. Finally, we discuss the role of STAT3 in the cardioprotection by ischemic and pharmacological/hypothermic conditioning and the effects of common risk factors and confounders on STAT3-mediated cardioprotection in cells, in animal models and in the human heart.

## STAT3 overview

To begin, we briefly consider STAT3 structure, evolutionary conservation of *stat genes*, their regulation and the subcellular STAT3 localization. For a more detailed description the readers are kindly redirected to recent reviews [10, 60, 223].

### Structure of STAT3

STAT3 together with STAT 1-2-4, STAT5a-b, and STAT6 are members of the STAT family. These proteins exhibit different functions while having an almost similar structure. This family of signal molecules appeared early in evolution and differentiated during the evolution of animal species. During animal development, the STAT signaling pathway regulates cell fate decisions. Comparative genomics displayed multiple duplications of the *stat gene* that occurred throughout the evolutionary history of metazoans. Many of these STAT duplications evolved into new genes through rapid sequence differentiation and the acquisition of new functions. It appears that the regulatory networks of *stat genes*, which include *stat1, 4, 5* and *stat6*, appeared early in vertebrate evolution. Among the STAT genes, the gene encoding STAT3 is the only one that is essential for the early development of vertebrate embryos and results in embryonic lethality if it is completely deleted [207]. In mammals, the two *stat5 genes* likely arose from a duplication event in early Eutherian evolution, a period from about 310 to 130 million years ago when the avian-mammal divergence and the separation of marsupials from other mammals occurred [60, 223]. These comparative analyzes indicate that genome-wide duplications and gene duplications for unbalanced chromosomal crossings were probably the main mechanisms underlying the evolution of STATs [223]. The selective persistence of the basic domains required for phospho-tyrosine signaling, as well as the Src homology-2 (SH2) domain and a site for protein tyrosine phosphorylation, suggest the importance of these modules in all multicellular organisms. It is, therefore, of particular interest that the different proteins of the STAT family have acquired additional functions, apparently independent of phospho-tyrosine, without losing their participation in the basic signaling mode [61, 121, 225].

Members of the STAT family interact with janus kinase (JAK) proteins, including JAK1, JAK2, JAK3 and tyrosine kinase 2 (TYK2) [12]. For details on this interaction, the reader is redirected to a previous review [129].

**Fig. 3:**
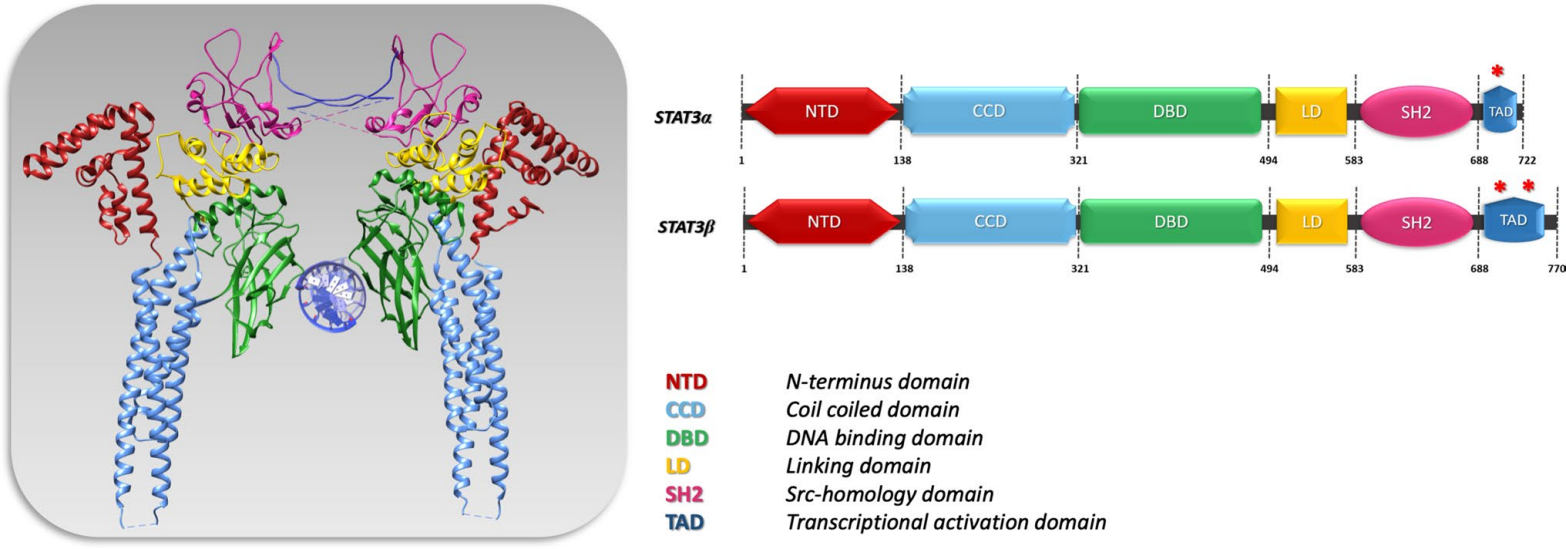
Structural representation of STAT3 dimer bound to a DNA molecule (molecular graphic performed with UCSF Chimera) and schemes of STAT3α and STAT3β domains. Red asterisks represent Y705 and S727 phosphorylation sites. *DNA* Deoxyribonucleic acid, *STAT3* Signal Transducer and Activator of Transcription 3, *UCSF* University of California, San Francisco

The human STAT3 protein (89 kDa), encoded by the *stat3 gene*, is constituted by 770 amino acids that are organized in 6 different domains [111] (Fig. 3). STAT3 contains an N-terminal oligomerization domain (OLG), whose role is still unclear; it does not seem to be involved in the formation of phosphorylated STAT3 homodimers nor in the interaction with DNA, but is suggested to play a role in non-canonical STAT3 signaling and in the formation of unphosphorylated STAT3 dimers [44]. Close to the OLG domain is the coiled-coil dimerization domain, which is involved in the interaction of STAT3 with regulators and other transcription factors. STAT3 comprises also a DNA binding domain (DBD) that is able to interact with the interferon γ-activated sequence (GAS) in the promoter of specific genes; the interaction with the DNA is mediated by four loops of STAT3, three loops belong to the DNA binding domain and one to the linker domain [13]. The linker domain also cooperates with the SH2 domain in the recognition of YXXQ sites. The SH2 domains recognize phosphorylated tyrosine residues of activated STAT3, allowing the formation of STAT3 homodimers that are essential in the canonical STAT3 signaling. The SH2 domains mediate the recognition of other phosphorylated tyrosine residues, for example on glycoprotein 130 receptor (gp130) [222]. Although the SH2 domain is highly conserved in the STAT family, it was possible to design specific inhibitors of STAT3 targeting the SH2 domain, such as Stattic or S3I-201 [41].

At the C-terminus, the transcription activation domain (TAD) can be detected with two phosphorylation sites, Tyrosine 705 (Y705) and Serine 727 (S727). The STAT3 phosphorylation at these two sites will be recognized by a specific pocket in the SH2 domain of the other STAT3 monomer; therefore, TAD domain participates in the stability of the homodimer and to activation of STAT3 signaling. For the recognition of Y705 and S727, the SH2 domain of STAT3 requires the presence of arginine, R609 that is highly conserved in all SH2 domains [184]. There are two main isoforms of STAT3 (α and β), which differ in the length of the C-terminus: the most abundant STAT3α comprises 770 amino acids and the less frequent STAT3β, which lacks 48 amino acids of the TAD, including the S727 phosphorylation site (Fig. 3) [184].

STAT3 canonical functions are primarily described as translocation from cytoplasm to the nucleus, where it acts as a transcription factor. However, STAT3 acts in non-canonical ways outside from the nucleus, by regulating the functions of mitochondria, endoplasmic reticulum (ER) and lysosomes. Yet, co-localization of cytoplasmic STAT3 with a microtubule-destabilizing protein *stathmin* favors microtubule polymerization and cell movement [158].

Effects of STAT3 on mitochondrial function are described below (see “Effects of STAT3 on mitochondrial function”). Here, we briefly describe the role in lysosomes and ER. STAT3 associates with lysosomes, spherical vesicles whose lumen's acidic pH (~ 4.5–5.0) is optimal for the enzymes involved in hydrolysis of different biomolecules. In lysosomes, STAT3 stimulates V-ATPase activity and contributes to the preservation of the acidic lysosomal lumen and the alkaline cytosol [135]. The ER is the principal Ca^2+^ storage compartment and its close contact with mitochondria membrane determines mitochondrial Ca^2+^ responses in many cell types [176]. STAT3 phosphorylation at Y705 and S727 residues (see below “Phosphorylation at Y705 and S727”) are not required for either ER localization or Inositol 1,4,5-trisphosphate Receptor (IP3R) type 3 (IP3R3) interaction. However, the phosphorylation on S727 may play an important regulatory role, as murine embryonic fibroblast cells expressing a STAT3 mutant on S727 showed excess Ca^2+^ release and apoptotic cell death after treatment with H_2_O_2_ or the intracellular ROS generator menadione, which induce cell death by apoptosis. These effects were not observed in wild type cells. Furthermore, ER STAT3 acts by promoting the proteasomal degradation of IP3R3, probably through the ubiquitin E3 ligase FBXL2 in mammalians [9, 103]. These observations are consistent with the fact that some breast cancers show an inverse correlation between phospo-STAT3 and IP3R3 protein levels [9].

## Post-translational modifications of STAT3: focus on the SAFE pathway

STAT3 post-translational regulations include phosphorylations and alternative post-translational modifications.

Apart from the phosphorylation of Y705 and/or S727 residues, STAT3 is reported to undergo alternative post-translational modifications, such as K685 acetylation, or C259 nitrosylation, and other modifications in non-canonical pathways. Here, for space constraint, we choose to consider mainly Y705 and S727 phosphorylation, which are pivotal in the SAFE pathway. All post-translational modifications are described and summarized in Table 1; for further information about STAT3 post-translational modifications the reader is redirected to previous reviews [66, 251].

**Table 1:** Post-translational modifications of STAT3

Post-translational modifications	STAT3 site	Effects	Reference
Phosphorylation	Y705	Dimerization and translocation of the protein into the nucleus and constitutes the canonical pathway of STAT3 activation	[249]
	Y705, S727	Promotes oncogenic activity of RhoA	[11]
	S727	Negatively modulates STAT3 Y705 phosphorylation	[36]
	S727	Extends the function of mTOR, including transcriptional regulation	[239]
	S727	Inactivates activated STAT3 leading to both pY705 dephosphorylation and post-activation nuclear export	[234]
	Y705, S727	Stimulates respiration and inhibits calcium-induced MPTP opening, contributing to cardioprotection	[20]
	S727	Interacts with GRIM-19 to facilitate STAT3 mitochondrial localization	[210]
	S727	Activates complex I and II	[225]
	S727	Increases mRNA for complex I subunits and decreases ROS generation	[243]
	Y705	Increases mitochondrial respiratory capacity and biogenesis	[1]
	Y705, S727	Regulates ER Ca2 + homeostasis	[9]
	S727	Regulates mitochondrial metabolism	[201]
	S727	Moderates ROS levels in the post-ischemic phase	[229]
	Y705	Reduces infarct size in LAD-ligated pig hearts	[100]
	Y705	Increases Ca2 + retention capacity, preserves complex 1 respiration and reduces infarct size	[79]
	S727	Attenuates myocardial IR injury	[250]
	Y705	Regulates cardiomyocyte survival and remodelling	[49]
	Y705	Reduces infarct size in LAD-ligated pig hearts	[191]
	Y705	Reduces infarct size and improves lefte ventricle (LV) diastolic pressure	[82]
	Y705	Reduces IR injury in isolated rat hearts	[38]
	Y705	Involved in efferent vagal nerve activation and spleen stimulation, releasing humoral cardioprotective substances	[133]
	Y705	Less vacuolization of mitochondria in the children undergoing open heart surgery and RIPreC attenuating myocardial IRI	[230]
	Y705	Determines hypothermia-induced protection in H9c2 cells	[88]
	Y705	Reduces infarct size in LAD-ligated rat hearts	[45]
	Y705	Reduces infarct size in LAD-ligated rat hearts	[62]
	Y705, S727	Anti-apoptotic effects on cardiomyocytes	[187]
	Y705	Ameliorates the contractile force in human atrial trabeculae exposed to H/R	[125]
	Y705	Reduces infarct size in *in-vivo* mouse hearts	[7]
	Y705	Reduces infarct size in *in-vivo* mouse hearts	[160]
	Y705	Attenuates cardiac fibrosis by regulating the macrophage polarization	[123]
	Y705	Reduces infarct size in isolated rat hearts	[193]
	Y705, S727	Improves LV contraction and decreases expression of pro-apoptotic proteins	[47]
	Y705	Reduces IR injury in *ex-vivo* and *in-vitro* models	[53]
	S727	Reduces IR injury in isolated mice hearts	[197]
	S727	Regulates mitochondrial respiration in *in-vivo* model	[220]
	S727	Mediates cardioprotection as a modulator of ETC activity in the mitochondria	[206]
	S727	Reduces infarct size in mice	[18]
	Y705	Reduces hypertrophy in rat model	[29]
	S727	Attenuates diabetic rat heart IR injury	[217]
	Y705	Limits diabetic cardiomyopathy in *in-vivo* and *in-vitro* models	[203]
	S727	Reduces infarct size in diabetic rats	[131]
	Y705	Reduces infarct size in rats	[124]
Acetylation	K49, K87 (N-terminus) and K685 (SH2 domain)	Enhances STAT3-mediated gene transcription and protects cardiomyocytes *vs* doxorubicin-oxidative stress	[91]
	K370, K383	Improves mitochondrial morphology and function in neonatal mouse cardiac myocytes	[156]
	K685	Induces Y705 phosphorylation transcriptional activity of STAT3 by increasing and dimer stability in prostate cancer cell line (PC3)	[241]
	K87	Favors the translocation and mitochondrial functions of STAT3 in starved cancer cells	[232]
Methylation	K140 dimethylation	Decreases STAT3-dependent transcription in A4 cancer cells	[233]
	K180 trimethylation	Stimulates STAT3 phosphorylation and transcriptional activity in glioblastoma and prostate cancer cells	[95]
Sulfydration	no direct sulfydration detected on STAT3	Activates STAT3 by Y705 phosphorylation and protects cardiomyocytes vs doxorubicin-oxidative stress	[228]
S-nitrosylation	C259	Blocks Y705 phoshorylation in microglial cells	[96]
Ubiquitination	K97	Induces the recruitment of BRD4 and the transcription of anti-apoptotic genes in HepG2 cells	[174]

### Phosphorylation at Y705 and S727

It seems that, while the Y705 phosphorylation of STAT3 is necessary for homodimerization and translocation in the nucleus, S727 phosphorylation is necessary for its translocation into the mitochondria [78, 79, 225]. Data suggest that a dynamic balance exists between the phosphorylation of Y705 and S727, which is achieved through a possible inhibitory effect of phosphorylated S727 on phosphorylated Y705 [11, 39, 239]. Nevertheless, the results obtained are still discordant and the role of phosphorylation in cardiovascular tissues is still not well defined.

Phosphorylated STAT3 belongs mainly to the JAK/STAT signaling pathway that is a multifunctional pathway that regulates immunity, cell division, cell death and cell differentiation [173]. Different receptors can activate this pathway, including the Erythropoietin, Leptin and Angiotensin II receptors, as well as the Tumor Necrosis Factor Receptor 2 (TNFR2) and Gp130. The latter is a common receptor for the cytokines of the Interleukin 6 (IL-6) family including IL-5, IL-6, IL-11, Oncostatin M, Leukemia Inhibitory Factor, and Cardiotrophin 1. Upon ligand binding, the receptor undergoes multimerization that brings JAK proteins associated with the receptor cytoplasmic tail close together, allowing trans-phosphorylation. The activated JAK proteins phosphorylate the YXXQ sites on the receptor's cytoplasmic domain. These sites are recognized by the SH2 domain of STAT3, which will be in turn phosphorylated by JAKs on Y705 of the TAD.

The phosphorylation of Y705 is the crucial event needed to induce the dimerization of STAT3. STAT3 dimers move into the nucleus and regulate the transcription of different genes through the DBD. Y705 phosphorylation of STAT3 occurs as consequence of the stimulation by various cytokines, such as IL-6 and Interferon γ (IFNγ), as well as growth factors, such as Fibroblast Growth Factor and Epidermal Growth Factor (EGF) [249]. Therefore, the induction of these pathways stimulates the phosphorylation of Y705, which subsequently leads to dimerization and translocation of the protein into the nucleus and constitutes the canonical pathway of STAT3 activation (Fig. 2).

It has been reported that mitogen-activated protein kinase (MAPK) family members including extracellular receptor kinase 1/2 (ERK1/2), c-Jun N-terminal kinase (JNK), and p38 can phosphorylate STAT3 at S727 [66]. Intriguingly, ERK1/2 is activated upon growth factors and cytokine stimulation and provides cardioprotection in IR settings via the RISK pathway activation [213]. Yet, JNK and p38 are known to be activated upon stress conditions and inflammatory cytokines, such as TNFα, which is also involved in SWOP [40] and SAFE (see below,  “STAT3 in the SAFE Pathway”). Interestingly, p38 is known to be a mediator of IRI, while JNK may have a dual effect, since evidence of JNK participating in myocardial damage [175] or in cardioprotection [185] have been reported.

Other serine/threonine kinases that are able to phosphorylate S727 are protein kinase C (PKCε and PKCδ isoforms), mammalian target of rapamycin (mTOR) and cyclin dependent kinase 5 (CDK5) [252]. Of note, the activation of PKCε before ischemia protects mitochondrial function and reduces apoptosis, mimicking IPreC, whereas it seems that the inhibition of PKCδ during reperfusion protects the heart from IR injury. Nevertheless, conflicting studies have suggested that PKCδ activation plays a protective role in IR injury and that PKCδ knockout (KO) mice exhibit increased myocardial ischemic injury [146]. Moreover, it has been suggested that the activation of PKCδ before ischemia triggers a protective effect against myocardial IR alterations in both isolated perfused rat hearts and cultured cardiomyocytes [4].

In cancer cells, S727 phosphorylation has been observed during the canonical STAT3 activation, where it may have a role in enhancing the transcriptional activity of STAT3, for example by recruiting cofactors, such as the histone acetyltransferase p300/CBP or by promoting the homodimer formation [3]. Yet, some works have shown that phosphorylation of S727 drives a reduction of the levels of Y705 phosphorylation in cancer cell lines. Yang et al. [234] demonstrated that the phosphorylation of S727 triggers the dissociation between pY705 and the SH2 domain of STAT3, thereby disrupting dimer formation. The phosphorylation of S727 enhances the dephosphorylation of Y705 by recruiting phosphatase TC45, therefore, limiting the duration of STAT3 transcriptional activity in a human liver cancer cell line (HepG2) [234]. Similar studies are lacking in cardiac cells.

### Acetylation

In response to stimulation by growth factors and cytokines, STAT3 can also be acetylated on multiple lysine residues (K) by histone acetyltransferase CBP/p300. Acetylation at K49, K87 (N-terminus) and K685 (SH2 domain) enhances STAT3-mediated gene transcription and protects cardiomyocytes from doxorubicin (DOXO)-oxidative stress [91]. It has been also observed that acetylation in K87 may favor the translocation in mitochondria of STAT3 in starved cancer cells after serum reintroduction or insulin stimulation [232]. Yet, acetylation in K685 induces the transcriptional activity of STAT3 by increasing tyrosine phosphorylation and dimer stability in prostate cancer cell line (PC3) [241]. STAT3 acetylation is also involved in the improvement of mitochondrial morphology and function in neonatal mouse cardiac myocytes [156]. It has been shown that after resveratrol treatment, STAT3 acetylated in K685 can silence tumor suppressor genes by recruiting DNA methyltransferase 1 to their promoters [120]. Yet, the nicotinamide adenine dinucleotide (NAD)-dependent silent information regulatory protein (SIRT) 1 can deacetylate STAT3 in the liver [159]. Of note, SIRT1-induced deacetylation leads to reduced mitochondrial localization and function of phosphorylated STAT3 in murine embryonic fibroblast cells [15].

### Methylation

In the nucleus, STAT3 methylations have been described on the K140 or K180 residues, with different effects on STAT3 activities. Histone methyltransferase SET9, a histone-modifying enzyme, is capable of methylating STAT3 on K140, leading to modulation of transcriptional activation of target genes in A4 cancer cells [233]. On the other hand, the enhancer of zeste homolog 2 (EZH2) component of the polycomb 2 complex determines K180 tri-methylation, which is essential for maintaining STAT3 phosphorylation and transcriptional activity in glioblastoma and prostate cancer cells [90]. The majority of studies on acetylation and methylation have been performed on mammalian cancer cells.

### Glutathionylation, oxidation, S-nitrosylation and sulfhydration

STAT3 can be oxidized, S-nitrosylated, sulfhydrated and glutathionylated on multiple cysteine residues, suggesting that STAT3 controls redox homeostasis and ROS signaling induced by cytokines and growth factors. Using antioxidants in studying the effects of cytokines on cells or in stimulating cells (fibroblasts and A-431 carcinoma cells) with H_2_O_2_, ROS have been shown to play a signaling role in the control of JAK, STAT3 and STAT5 [188]. The activation of STAT3 and STAT5 by ROS upon cytokine or growth factor treatment can also determine the activation of NADPH oxidase (Nox) in human aortic smooth muscle cells [92] and in cancer cells [240]. In particular, ROS inhibit STAT3 transcriptional activity, either downstream of the IL-6 signal or in conditions of oxidative/nitrosative stress [107, 132, 196, 231]. Of note, moderate ROS production downstream of growth factors such as insulin-like growth factor 1 (IGF1) and EGF can activate JAK kinases and enhance STAT3 phosphorylation and its nuclear activity [35, 110, 122, 188]. STAT3 oxidation can be observed downstream of cytoplasmic thiol peroxidase peroxiredoxin-2 (Prx2) [196]. This limits ROS toxic effects, but impairs IL-6-induced and STAT3-mediated transcription. STAT3 sulfhydration favors STAT3 activation by Y705 phosphorylation and protects cardiomyocytes from DOXO-oxidative stress [228]. Finally, S-nitrosylation at C259 blocks Y705 phoshorylation in microglial cells [96] and ubiquitination at K97 induces the recruitment of BRD4 and the transcription of anti-apoptotic genes in HepG2 cells. Thus, the inhibition of the STAT3 mono-ubiquitination–BRD4 pathway may be useful for the treatment of STAT3-dependent tumors [174].

All in all, it appears that a cross-talk between oxidative and non-oxidative modifications of STAT3 can influence canonical and unconventional STAT3 activities, including cell proliferation and survival. The complex functions of STAT3 in redox homeostasis have recently been reviewed [8, 102, 134].

### STAT3 in the SAFE Pathway

Post-translational modification of STAT3 is a pivotal step in the SAFE pathway in which TNFα and its receptors play key roles. As said this pathway can be activated in conjunction with the RISK pathway and it has been proposed to play a more important role in larger mammals [70, 79, 189, 190]. Here we point out some aspects of SAFE in relation to STAT3.

First, it has been observed that in rodent hearts TNFα protects against IR in a dose-dependent manner [43, 112, 117]. Besides TNFα, other agonists have been suggested in SAFE signaling, including high density lipoprotein (HDL), sphingosine-1-phosphate (S1P), IL-10, insulin and stromal cell-derived factor-1 alpha [26, 27, 46, 52], to name a few. Furthermore, TNFα emerged as an important cardioprotective endogenous factor released by cardiomyocytes during IPreC and IPostC maneuvers [113, 118]. Two isoforms of the TNFα receptor have been described in the heart, the TNFR1 and the TNFR2. Intriguingly, exogenous TNFα induces cardioprotection in TNFR1 KO mice, but is unable to protect TNFR2 KO mice, thus suggesting that cardioprotection is mediated by TNFR2 activation [114]. In addition, it appears that TNF-receptor-associated-factor 2 (TRAF2) is the downstream target of TNFR2 in mice [24]. Yet, TRAF2 is able to activate the formation of the S1P via sphingosine kinase 1 (SK1) activity [5] and the protective effect of TNFα is blunted in the presence of the sphingolipid pathway inhibitor, N-oleoylethanolamine [28]. These data suggest that S1P can act as a downstream target of TNFα/TNFR2/TRAF2 to confer cardioprotection [117]. TNFα binding to its specific receptors activates the JAK-STAT3 pathway. In particular, JAK2 phosphorylates and makes a docking site for STAT3 which, after phosphorylation is activated (Fig. 2). Of note, it has been proposed that TNFα is able to induce Y705 phosphorylation even if the interaction between TNFR2 and JAK2 is still uncovered [63]. Indeed, this interaction could result in the activation of intracellular SK1 and S1P production in cell lines [5]. Following the binding of TNFα to TNFR2, the activation of TRAF2 could upregulate SK1, which in turn could catalyze the formation of intracellular S1P and the subsequent activation of JAK-STAT3 (Fig. 2).

In the next chapter, we analyze the mitochondrial function affected by IR and the role of STAT3 in affecting mitochondria function. Indeed, mitochondrial targeting is essential in the SAFE pathway.

## Mitochondrial functions affected by IR

Before to analyze the mitochondrial STAT3 role we briefly describe the role of mitochondria in IRI (for more details the reader is redirect to other review, e.g., [69, 76–78, 162, 164]*,*).

Under physiological conditions, mitochondria guarantee energy requirements, while under stressful conditions, they represent the judges of cell fate between life and death [128]. The heart is a high-energy-consuming organ as cardiomyocytes contain a large number of mitochondria (about 30–40% in volume). Therefore, the heart will be heavily affected by the inefficient supply of oxygen and the consequent alteration of the mitochondrial status.

*During ischemia,* the metabolism of myocardial tissue, initially mainly aerobic, turns towards an anaerobic phenotype. The mitochondrial metabolism is compromised by a drop in intracellular pH and a strong reduction in the ATP production [74]. ATP depletion causes a sensible reduction of the activity of Na^+^/K^+^-ATPase, and other ion pumps fundamental for the maintenance of the physiological mitochondrial membrane potential (∆Ψm), including the Na^+^/H^+^ exchanger, the Na^+^/Ca^2+^ exchanger and the L-type Ca^2+^ channel [202]. In particular, the activation of L-type Ca^2+^ channel contributes to Ca^2+^ overload, with subsequent activation of nucleases, proteases and phospholipases. Another crucial event characterizing both *ischemia and reperfusion* is the ROS production, which derives from altered activity of both ETC and enzymes, such as xantine oxydase (XO), NADPH oxidase, cytochrome P450 oxidases, uncoupled nitric oxide synthase (NOS), and monoaminooxidase (MAO) [42]. All of these changes lead to depression of respiratory chain complex activity, inappropriate oxidative phosphorylation and ATP synthesis by F1F0-ATPase, that in turn determine a reduction in ∆Ψm, an additional Ca^2+^ overload, the ROS-induced ROS release (RIRR) phenomenon, mitochondrial swelling and cytochrome *c* release promoting cell death [180, 202].

*Upon reperfusion*, and in particular immediately in the first phase, compared to ischemia, ROS production may lead to the peroxidation of mitochondrial respiratory complexes, including the SH groups of complex I and cardiolipin, which is needed for complex III and complex IV activity. In IR setting the F1F0-ATPase not only reduces the production of ATP but may also reverse its operation by hydrolyzing the ATP present as the proton motive force that pushes the ETC is dissipated [143]. Therefore, the loss of ATP during IR is further exacerbated [67]. Of note, F1F0-ATPase pump is recognized as the main component of mPTPs [14]. Despite the controversy surrounding the components that make up mPTPs, there is greater consensus on the triggers that induce their opening. Conditions such as Ca^2+^ overload, excessive ROS production, ATP depletion and an increase in inorganic phosphate contribute to a large extent to their opening [162]. mPTPs opening is favored by pH recovery during reperfusion [89] with a subsequent collapse of the inner membrane, decoupling of the respiratory chain, arrest of mitochondrial ATP synthesis, and ultimately mitochondrial swelling, rupture, and cell death. The pharmacological inhibition of mPTPs opening at the beginning of reperfusion can attenuate cardiomyocyte death and reduce myocardial IS [25, 162]. Of note, F1F0 ATPase and mPTPs may also have a role in triggering IPreC [68, 169].

As said above and as reported in Fig. 1, three main signaling cascades that converge to mitochondria have been identified in the context of IPreC and IPostC. It has been proposed that glycogen synthase kinase-3β (GSK-3β) acts directly on components on the mPTPs as well as on Bcl-2 family proteins [164]. Moreover, the cGMP-PKG pathway may lead to the phosphorylation of mitochondrial PKCε1, which promote the opening of the mitochondrial ATP-sensitive K^+^ channel (mitoK_ATP_) [55]; once opened, these channels allow increased uptake of K^+^ in the matrix, leading to alkalinization of the mitochondrial membrane. To counterbalance this event, Complex I of ETC increases its activity and produces more superoxide and its products H_2_O_2_ and hydroxyl anion radical. The increased ROS are able to activate another PKC, PKCε2, which in turn will inhibit the mPTPs with a phosphorylation-dependent reaction, thereby preserving mitochondrial functionality [164].

Mitochondria are dynamic organelles that continually change their shape by undergoing quality control mechanisms, that include mitochondrial fission, fusion and mitophagy, a specialized form of autophagy; these are fundamental processes to maintain mitochondria turnover and assure metabolic need of cardiomyocytes [76, 162]. Upon IR stressful conditions, mitochondria may activate these quality control mechanisms. Thus removing defective mitochondria and restoring the energetic balance, to protect cardiomyocytes against IRI. Mitophagy should be finely regulated to maintain a favorable balance between dying mitochondria and new functional mitochondria, which should restore ATP production. During IR excessive mitochondrial fission may lead to cardiomyocyte death. In particular, dynamin related protein-1 (DRP1), the regulator of mitochondrial fission, is able to recruit Bax/Bak and promotes the mPTP formation at the outer mitochondrial membrane, triggering cytochrome c release. In addition, mitochondrial fusion may provide a protective effect during IR, by counterbalancing the excessive mitochondrial fission, and equilibrating mitochondrial proteins and mitochondrial DNA (mtDNA) [104]. In this context, STAT3 may play a pivotal role (Fig. 2) as we describe below (see also “Effects of STAT3 on mitochondrial quality control”).

## Effects of STAT3 on mitochondrial function

STAT3 non-genomic functions raise still some questions about how, where, and when its action is exerted. In particular, in cardiac IRI models, mitochondria are the most interesting target of the investigation, since they are highly represented and can determine the life and death of cardiac cells. Besides mitochondrial quality control, ETC, ΔΨm, Ca^2+^ regulation, redox balance, and mPTPs status are promising targets that can be affected by STAT3 non-genomic function.

*Localization of STAT3 in mitochondria.* To investigate the role of STAT3 in mitochondria, it is necessary to verify its localization and how it is redirected and translocated in mitochondria. Even before the discovery of the SAFE pathway, several research groups studied the interaction of STAT3 with candidate proteins and their localization in mice and rats cardiomyocytes [65, 93, 229]. The interest started in the contest of cancer research, focusing on finding molecules able to interact and inhibit the transcriptional activity of STAT3, which is known to regulate cell growth and apoptosis and is constitutively active in various cancers [119]. Even if the cellular models are different, the identification of interactors of STAT3 has been the first step to understand the mechanisms promoting the mitochondrial localization of STAT3 in cardiac cells. Several evidences obtained with techniques, such as Yeast two-hybrid, co-immunoprecipitation, and immunofluorescence, suggest that the main mitochondrial interactor of STAT3 is gene associated with retinoid interferon-induced cell mortality 19 (GRIM19) [140].

*Phosphorylation of mitochondrial STAT3*. Zhang et al. [244] found that the mutagenesis of S727 into alanine determines the suppression of the interaction with mitochondrial GRIM19 in HeLa cells. Therefore, phosphorylation of S727 sites has been proposed as necessary for mitochondrial localization (Fig. 2). Nevertheless, the timing and nature of STAT3 phosphorylation should still be determined, to understand if the phosphorylation occurs outside or inside the mitochondria and if phosphorylation of S727 is fundamental in determining the import in mitochondria. Boengler et al. [20], among others, found that STAT3 phosphorylated at both Y705 and S727 and co-immunoprecipitated with translocase of the outer membrane 20 (TOM20) in mitochondria extracted from the rat left ventricle.

*Import into mitochondria and interacting mitochondrial proteins*. Zhang et al. [244] showed that GRIM19, in different cancer cell lines, interacts specifically with STAT3 but not with other STAT proteins, such as STAT1 and STAT5A. This selectivity may be due to the differences that STAT family members bear in the TAD, which deletion prevents the co-immunoprecipitation of these two proteins. Although the aim of Zhang et al. [244] was to demonstrate that GRIM19 was able to inhibit the genomic function of STAT3 in the tumorigenesis progression, subsequently Tammineni et al. [210] found that STAT3 is enriched in the inner mitochondrial membrane isolated from rat heart and suggested that GRIM19 influences STAT3 integration into complex I. Indeed, GRIM19 was found to promote STAT3 import into mitochondria and to favor STAT3 integration into complex I. As said, these two events may depend on the S727 site of STAT3 C-terminus. Indeed, in mitochondria from rat hearts, the deletion of the C-terminus and the mutation of serine 727 into alanine (S727A) abolished the import of STAT3 and the assembly into complex I [210]. Even if STAT3 lacks the mitochondrial targeting sequence required for the mitochondrial import, TOM20 has been proposed to recognize STAT3 and mediate its translocation inside the mitochondria [20].

Despite these evidences, some studies did not find STAT3 in the mitochondrial fraction of mice hearts [65]. Some of these works investigated the hypothesis that STAT3 may exert its non-genomic function in other non-nuclear sites, such as in the ER. For instance, Su et al. [201] demonstrated that STAT3 is present in the mitochondrial-associated ER membrane (MAM) of different tissues (liver, lungs, brain). A data confirmed by Avalle et al. in cancer cell lines [9] (see below “Effects of STAT3 on Ca^2+^ regulation”).

### Effects of STAT3 on mitochondrial ETC

As mentioned before, mitochondrial damage occurring during ischemia can be partially explained by the alteration of ETC functionality, followed by enhanced ROS generation during reperfusion [205]. It is possible to induce cardioprotection by targeting ETC complexes and regulating mitochondrial respiration. STAT3 has been shown to interact with ETC complex I and II, preserving their activity in mitochondria from mice hearts and enhance ATP production in human cancer cells [61, 225]. As result, aerobic cellular respiration would be optimized, and ROS formation would be under control (Fig. 2). Particularly, Wegrzyn et al. [225] considered the role of STAT3 in cellular respiration. First, they verified the mitochondrial and submitochondrial location of STAT3 in cells isolated from mouse heart, brain, kidney, spleen as well as human cell lines. One-tenth of the cellular STAT3 has been found in the mitochondrial fraction of these cells. STAT3 and GRIM19 resisted the proteinase K treatment of isolated mitochondria, indicating that they may be located in the inner membrane or the matrix, rather than in the outer membrane. STAT3 co-immunoprecipitated with components of complex I and II, which activity was then measured in the absence and presence of STAT3. Cardiomyocytes from STAT3-deficient mice showed a decreased rate of O_2_ consumption as well as reduction of complex I and II activity, but not complex III. In STAT3-deficient pro-B cells the loss of complex I and II activity was restored by both full-length STAT3 and by MLS-STAT3, the mitochondrial form of STAT3. The latter is obtained by adding the mitochondrial targeting sequence of cytochrome c oxidase subunit VIII to STAT3. A mutagenesis study of MLS-STAT3 then revealed that the S727, but not the Y705 site was fundamental to mediate the activation of complex I and II [225].

However, it is still unclear whether the control of mitochondrial localization, complex I/II association, or other step might be responsible of the STAT3 regulation of the respiration [154]. Whether such a regulation pertains to STAT5 in different species is alternatively discussed [245].

Direct interactions between STAT3 and complex III and IV were not found; however, the existence of mitochondrial respiratory supercomplexes formed by complex I, III, and IV [128] does not allow to rule out the influences of STAT3 on these supercomplexes. Contradictory results are reported regarding the ability of STAT3 to decrease ROS generation by complexes I and III of the ETC in mitochondria from mice left ventricular tissue [21]. The low ratio between complexes I and II and STAT3 in mitochondria of porcine and murine hearts [170] is against the hypothesis that STAT3 might be a structural component of these complexes. Therefore, further analyses are required to determine the nature of the interaction between STAT3 and the proteins of the ETC.

Other studies investigated whether different molecules improve the recovery of mitochondrial function after IRI through STAT3 signaling within mitochondria. For instance, Zhang et al. [243], used Zinc either on isolated rat hearts or H9c2 cardiomyoblasts subjected to IR or Hypoxia/Reoxygenation (H/R), respectively. They observed that Zinc treatment leads to increased phosphorylation of STAT3 at S727 compared to Y705. Yet, the balance of cytosolic and mitochondrial STAT3 was in favor of the latter, suggesting that the phosphorylation of S727 could direct STAT3 towards mitochondria. Moreover, it has been shown that Zinc-induced S727 phosphorylation is involved in the enhancement of mitochondrial oxidative phosphorylation. Particularly, in isolated rat hearts exposed to IR, Zinc induced a sensible improvement of the rate of respiration, which determined an increased ATP level and citrate synthase activity. These data are supported by an increase in mRNA for complex I subunits and a decrease in ROS generation [243].

The mechanism by which STAT3 interacts with Complexes I and II is still unknown; there is contrasting evidence about whether mitochondrial STAT3 can modulate the levels of mRNA of these complexes, but in the majority of the cases, the mRNA levels are unchanged in presence or absence of mitochondrial STAT3. Intriguingly, STAT3 was found to bind mtDNA in several cell types and to interact with mitochondrial transcription factor TFAM in keratinocytes, where it regulates the level of some mitochondrial-encoded transcripts, such as NADH dehydrogenase 5/6 and cytochrome b [142]. Of note, the prevention of mtDNA degradation has proven to be cardioprotective in in vivo and ex vivo* rat* hearts [237]. Nevertheless, to the best of our knowledge, despite STAT3 binding to mtDNA, no evidence has been reported on the role of STAT3 in influencing mitochondrial-encoded transcript and/or mtDNA degradation in the context of cardioprotection. Further investigation is required to elucidate the exact pathway by which STAT3 affects mitochondrial ETC.

### Effect of STAT3 on ROS generation

The mitochondrial redox state is determined by the balance between the production of reactive species by the ETC and the other sources of ROS indicated above on the one hand and the scavenging of ROS by antioxidant enzymes on the other hand.

For instance, during IR events, ETC is altered and consequently, ROS production is increased and further sustained by the RIRR. This leads to cardiolipin peroxidation at the inner membrane that favors mPTPs opening and cytochrome c release from mitochondria, promoting apoptosis [212]. Studies in cardiomyocytes showed that DOXO treatment caused a reduction of the complex I in mitochondria, as well as increased production of ROS [247]. The cardiac-specific overexpression of STAT3 led to a decreased toxicity of DOXO in mice and in primary ventricular murine cardiomyocytes [106, 177], suggesting that STAT3 may be able to attenuate ROS formation, even if its precise mitochondrial function has not yet been discovered. Studies report a strong inhibition of complexes I and II in cardiac tissues [225] and a low membrane potential, a decreased ATP level and an increased ROS formation in the absence of mitochondrial STAT3 in astrocytes [181].

It is not clear how STAT3 can promote mitochondrial respiration and at the same time minimize ROS formation. Szczepanek et al. [205] speculate that during ischemia STAT3 is able to reduce the levels of superoxide produced by complex I, and to address, decreasing cardiolipin oxidation, the superoxide towards the mitochondrial matrix, thereby protecting the inner membrane and preventing the release of cytochrome c. This speculation was supported by the observation that cardiomyocytes overexpressing mitochondrial-targeted STAT3 had reduced ROS levels in comparison with wild type mitochondria upon ischemia. Additional evidence supporting that STAT3 is able to keep ROS under control is provided by Boengler et al. [20], who treated mitochondria extracted from rat left ventricles with the STAT3 inhibitor Stattic. In this case, the inhibition of STAT3 induced a dose-dependent increase in ROS formation from complex I, which was coupled with lower ATP production and Ca^2+^ retention capacity. Nevertheless, further research is needed as Stattic is not specific for mitochondrial STAT3 [183]. Moreover, we cannot rule out that STAT3 genomic function contributes to reducing ROS formation by altering the transcription of genes encoding scavenging proteins. An interesting study about the relationship between STAT3 and ROS is provided by Abid et al. [1]. They demonstrated that IL-6 exposure activates the canonical STAT3 pathway in myotubes favoring mitochondrial ROS formation and STAT3 Y705 phosphorylation, determining a sort of positive feedback between these two events. As a result, mitochondrial respiratory capacity and biogenesis were acutely increased. However, a decline in respiration and a further elevation in ROS and oxidative stress was observed when IL‐6 exposure was sustained [1].

### Effects of STAT3 on mPTPs opening

The most critical challenge to safeguard cardiac cell function during IR stress is to maintain the mitochondria integrity. As mentioned before, the opening of mPTPs is a crucial step leading to cell death and it occurs mainly during reperfusion of cardiac tissue, as a consequence of the damage caused by ROS and Ca^2+^ overload [108]. The point is discussed by Boengler et al. [20], who investigated the role of STAT3 in isolated mitochondria from cardiomyocyte-specific STAT3^−/−^ mice and in isolated rat heart mitochondria using the inhibitor Stattic. Mitochondria isolated from STAT3^−/−^ hearts undergo mPTPs opening upon exposure to lower Ca^2+^ levels compared to STAT3^+/+^ hearts [20]. Therefore, it can be hypothesized that STAT3^−/−^ mitochondria are more susceptible to reperfusion-induced damage when Ca^2+^ overload triggers mPTPs opening (Fig. 2). Indeed, IPreC and IPostC, which rely on the limitation of mPTPs opening at the beginning of reperfusion, do not trigger cardioprotection in mouse STAT3^−/−^ hearts [113, 194]. The reason behind this protective role of STAT3 is still unknown, but some possible hypotheses relate to the interaction of STAT3 with cyclophilin D (CypD), a mitochondrial protein identified as a regulator of mPTPs [151]. This binding is mediated by the amino terminus of STAT3, suggesting that other domains (amino acids 1–330 of STAT3) in mitoSTAT3 are relevant for its non-canonical mitochondrial role. The interaction STAT3-CypD occurs in the mitochondria with subsequent reduction of mitochondrial ROS production after oxidative stress [151]. As said above, another hypothesis is that STAT3 binds to the NTD of the mitochondrial Ca^2+^ uniporter (MCU) to reduce mitochondrial Ca^2+^ overload and thus ROS production in rat hearts and cardiomyocytes [229]. Nevertheless, a reduction of ROS formation may limit mPTPs opening and the RIRR phenomenon [212].

### Effects of STAT3 on Ca^2+^ regulation

Calcium regulation is one of the main events that denote the cell functionality; therefore, its concentration in different subcellular compartments is finely controlled. In the ER, Ca^2+^pump (SERCA), IP3R and their relative ryanodine receptors represent the gates that allow the entry and the release of Ca^2+,^ respectively [32], while in the mitochondria, Ca^2+^ concentration is important to regulate the activity of enzymes, the rate of respiration and to maintain mPTPs status. It has been proposed that STAT3 may have an important role in the closed relationship between ER and mitochondria. In this regard, Avalle et al. [9] showed that STAT3 interacted with IP3R3 on the ER and that its mutation in S727, but not in Y705, determined an excessive release of Ca^2+^ in the cytosol of breast cancer cell lines. They explained this observation by demonstrating that STAT3 promoted IP3R3 proteasomal degradation. Moreover, STAT3 silencing in STAT3-dependent MDA-MB-468 and -231 cells also significantly increased Ca^2+^ content and release from the ER [9]. On the other hand, in MDA-MB-453 or T47D cells, in which STAT3 is not active, the release of Ca^2+^ was not modified by the silencing of STAT3. Therefore, in STAT3-dependent cells, treatment with H_2_O_2_ or menadione increased Ca^2+^ release. It appears that STAT3 can regulate ER Ca^2+^ homeostasis when phosphorylated on both Y705 and S727 by localizing to the ER and MAM, where it physically interacts with the IP3R3 Ca^2+^ channels [9] (Fig. 2). IP3R is also known to mediate the interaction between MAM and voltage-dependent anion channels (VDAC) on the mitochondrial membrane [32]; therefore, is not excluded that STAT3, which has been found in the MAM context [201], may be involved in this cross-talk. In other words, the preservation of Ca^2+^ homeostasis by S727-phosphorylated STAT3 in the ER represents an indirect mechanism of mitochondrial metabolism regulation. Another evidence of STAT3 Ca^2+^ regulation is provided by Yang et al. [235], who examined the alterations of mitochondrial Ca^2+^ in STAT3^+/+^ and STAT3^−/−^ CD4 cells under IL-6 stimulation. They found that IL-6 promoted an increase in mitochondrial Ca^2+^ and that STAT3 was able to reduce its mitochondrial concentration, by promoting the Ca^2+^ export to the cytosol. This action was independent of the transcriptional activity of STAT3, but more likely elicited through mitochondrial STAT3.

It has been reported that MCU transports significant amounts of Ca^2+^ from the cytosol into mitochondria [51]. The activity of MCU may be influenced by STAT3. Indeed, it has been reported that during IPostC obtained with H_2_O_2_, STAT3 interacts with the N-Terminal Domain (NTD) of MCU in rat cardiomyocytes [229]. Moreover, a co-localization and interaction of STAT3 phosphorylated at S727 and MCU was observed in these cardiomyocytes. These results indicated that moderate ROS levels in the post-ischemic phase could activate STAT3 which inhibits the MCU via its interaction with the NTD of MCU to relieve mitochondrial Ca^2+^ overload [229].

### Effects of STAT3 on mitochondrial quality control

TNFR2 activation protects cardiac myocytes against stress by upregulating optic atrophy 1 (OPA1) expression. This process was facilitated by p300-mediated STAT3 acetylation and STAT3/RelA interactions, leading to improvements in mitochondrial morphology and function [156]. Yet, κ-opioid receptor activation promotes mitochondrial fusion and enhances myocardial resistance to ischemia and reperfusion injury via STAT3-OPA1 pathway [219]. Since mitochondrial quality control plays a vital role in cardioprotection, further investigations are needed to clarify the exact involvement of STAT3 in this process.

All in all, we can say that STAT3 plays a pivotal role in regulating mitochondrial function, fundamental organelles for the cardioprotection by conditioning procedures. Since STAT3 is central in the SAFE pathway, the activation of this pathway is necessary for the cardioprotective effect of ischemic conditioning in some species and conditions [19, 98, 101]. In the following Section we discuss these aspects.

## STAT3 in cardiac conditioning

As mentioned in the introduction, here we update the role that STAT3 plays in the context of different conditioning strategies, including IPreC, IPostC, pharmacological/hypothermic conditioning and RIC. The latter can be induced before (RIPreC), during (RIPerC) or after (RIPostC) the myocardial infarcting ischemia (Fig. 1). In all these procedures the involvement of STAT3 has been described. Indeed, TNFα or STAT3 cardiomyocyte KO are not protected by ischemic conditioning stimuli, but it is still possible to induce pharmacological conditioning [113, 118, 194, 195]. Therefore, some differences among procedures and species exist, as we will see, for example, considering the parallels between STAT3 and STAT5.

### STAT3 in ischemic preconditioning

As said, IPreC, defined as non-lethal episodes of myocardial IR applied before an ischemic event, is the first conditioning maneuver discovered more than 35 years ago [153]. Clinical evidence of the efficacy of IPreC in human hearts, supported by parameters, such as attenuated ECG changes, reduced lactate and creatine kinase release, are provided by the review of Heusch and Rassaf [81].

The comprehensive article of Kleinbongard [100] compared IPreC, IPostC and RIPreC in relation to the phosphorylation of different proteins involved in cardioprotection, including STAT3, Akt and ERK1/2. The phosphorylation of STAT3 in Y705 occurred at both 10 min and 120 min of reperfusion post left anterior descending artery (LAD) ligature only in pig’s hearts exposed to IPrec and not to IPostC nor RIPreC. Since any other signaling pathways were upregulated, STAT3 activation was associated with cardioprotective properties of IPreC in pigs.

The same result is confirmed by some other studies in pigs [57], but questioned by another study [200], in which the specific inhibition of ERK signaling and not STAT3, abrogated IPreC-mediated cardioprotection in pigs. Yet, Kwak et al. [109] reported that the protective mechanisms of sodium nitroprusside (SNP, 0.3 mM) preconditioning against high-concentration-SNP (1.5 mM)-induced apoptosis in H9c2 cardiomyoblasts was mediated by ERK1/2-STAT1/3 activation via PKC-dependent mechanisms. Indeed, in this model chelerythrine, a PKC antagonist, abolished the activation of both ERK1/2 and STAT1/3. Bolli et al. [23] used an inducible cardiomyocytes-specific STAT3 KO mouse to determine the role of STAT3 in mediating the IPreC-protection versus in vivo LAD ligation. In this study, IPreC upregulation of cardioprotective proteins, such as Heme oxygenase 1 and Cyclooxygenase-2 and antiapoptotic proteins (Bcl-xL and Mcl-1, c-FLIPL and c-FLIPS), was abrogated in cardiomyocytes-specific STAT3 KO mouse [23]. These experiments by Bolli and coll. [23] provide insight on the fundamental role of STAT3 in inducing cardioprotection in the late phase of IPreC and, therefore, its deletion abrogates all of these protective effects.

### STAT3 in ischemic postconditioning

IPostC refers to brief repeated cycles of ischemia and reperfusion that are applied at the ischemic site immediately after reperfusion [248]. IPostC may reduce IS, edema and improve left ventricle (LV) contractile properties in clinical studies [211]. However, other studies do not support these cardioprotective potentials (see below, “Some more considerations on mitochondrial STAT3 in IRI and cardioprotection” and “Effects of common risk factors and confounders on STAT3 cardioprotection”). 

The mechanisms of protection may be similar to IPreC, i.e., SAFE and RISK pathway, but IPostC protection may rely also on other pathways, which control redox state and pH, as well as on mitochondrial players influenced by STAT3. In this regard, Heusch et al. [79] studied the phosphorylation of STAT3 and the mitochondrial activity in pig hearts subjected to IR followed by IPostC. In isolated mitochondria from postconditioned hearts, STAT3 phosphorylation in Y705 was increased and they observed increased Ca^2+^ retention capacity and preserved complex 1 respiration in comparison with mitochondria from non-conditioned hearts. All these effects, including the decreased IS were reverted with the use of AG490, a specific and potent inhibitor of the JAK2/STAT3 pathway.

Zhu et al. [250] found that IPostC, in primary rat ventricular cardiomyocytes exposed to H/R, is able to reduce H/R-induced cell damage, with concomitant enhancement of STAT3 phosphorylation S727 but not at Y705. These authors were interested in the involvement of 5' Adenosine Monophosphate-activated protein kinase (AMPK) in IPostC and they found that STAT3 phosphorylation was not affected by the inhibition of AMPK, as well as the protective effects of IPostC were not abrogated, suggesting that IPostC in vitro relies on STAT3 and this is independent of AMPK signaling. In vivo model of MI achieved with LAD occlusion gave similar results with IPostC, increasing the level of S727 but not Y705 phosphorylation of STAT3 [250]. Moreover, they compared IPostC to pharmacological postconditioning with Adiponectin and they found that while IPostC induced STAT3 activation by S727 phosphorylation, the latter activated/phosphorylated STAT3 in Y705, supporting the difference between ischemic and pharmacological conditioning. Interestingly, the combination of IPostC and Adiponectin conditioning provided additional protection in comparison with the conditioning alone, and promoted the phosphorylation of both sites of STAT3 [250]. This suggests that the integration of canonical pathway of STAT3 (transcription of antioxidant and anti-apoptotic genes) with the mitochondrial pathway (reduction of ROS production, mPTPs opening prevention, optimization of complex I activity) is able to provide maximal cardioprotection in vivo.

### STAT3 in remote ischemic conditioning

Remote ischemic conditioning is performed by applying brief cycles of IR in a remote organ or tissue from the heart; it was demonstrated to protect the heart against subsequent ischemic events, not only in animal models, but also in humans and it is currently under studies in the clinic [33, 85, 171].

STAT3 signaling was activated in mice hearts subjected to LAD ligation, when RIPostC was applied in the left hind limb at the beginning of reperfusion [54]. Apoptosis and oxidative stress markers were decreased in the RIPostC group, but these protective effects were abrogated when mice underwent AG490 pre-treatment and STAT3 was inhibited. Accordingly, Billah and colleagues [16] studying the link between STAT3 and autophagy in H9c2 cells and rats, subjected to RIPreC and H/R and IR, respectively, found that RIPreC applied prior to IR reduced the IS and upregulated autophagy in heart tissue at 24 h post-RIPreC. Interestingly, they detected the activation of STAT3 and an increased plasma level IL-6 immediately after RIPreC, indicating that JAK/STAT3 signaling occurs upstream to autophagy upregulation. Besides, pretreatment with AG490 abolished RIPreC-induced autophagy upregulation in vitro. These results suggest STAT3-dependent autophagy activation as a putative mechanism for RIPreC cardioprotection. In another study with a similar model, the same group found that the RIPreC-induced increase in IL-6 plasma level and subsequent JAK-STAT pathway activation, was dependent on the early growth response-1 (Egr-1) transcription factor [17]. The silencing of Egr-1 led to a decrease in STAT3 Y705 phosphorylation and an increase in hypoxic damage in vitro and IS in vivo. Both these two studies highlight the importance of the increase of plasma IL-6 secretion after RIPreC to protect the heart against IRI. Yet, it has been proposed that IL-6 can signal in cardiomyocytes independently of STAT1/3 and that the activation of ERK1/2 and PI3K by IL-6 is required for inducing cardioprotection. Consistently, IL-6 induces the activation of MAPKs and in particular of p38-MAPK, which acts as negative feedback regulator of JAK/STAT activation in cardiomyocytes [49]. Indeed, the inhibition of p38-MAPK enhances STAT3 phosphorylation. This suggests that a balance exists between MAPKs and JAK/STAT in cardiomyocyte survival.

An interesting study of Skyschally et al. [191] focused on RIPerC and its mechanism of protection in pigs, rats and mice. RIPerC in pigs exerted a cardioprotective effect, whose magnitude was similar to the one induced by RIPreC [190, 191]. In both experiments, the conditioning maneuvers provided the same results in terms of protection by activating STAT3 but not RISK pathway in pig hearts, as indicated by low levels of Akt and ERK1/2 phosphorylation. The transfer of plasma from pigs who underwent either RIPerC or RIPreC, to isolated mice or rat hearts, protected these hearts from the ex vivo IR. Intriguingly, the transfer of humoral factors contained in the pig plasma, activated both RISK and SAFE pathways in rodent’s hearts and both pathways were fundamental to reduce IS. In isolated rat cardiomyocytes, the transfer of pig plasma helped to preserve complex I mitochondrial respiration, improve mitochondrial ATP production and reduce ROS formation. These effects were abolished by the use of both RISK and SAFE pathway inhibitors. To investigate whether RIC induces the release of cardioprotective factors also in humans, Hildebrandt et al. [82] harvested the plasma from healthy volunteers undergoing RIC from 5 min post procedure up to 6 days. The plasma was subsequently given to isolated mice hearts before the induction of IR and revealed to be protective and reduce IS up to 50%. An increased STAT3 phosphorylation, but not Akt and ERK1/2, was detected in mice hearts treated with the RIC-plasma and the additional treatment with STAT3 inhibitor Stattic abrogated the cardioprotection. The above studies suggest that STAT3 is crucial in pigs and humans, whereas RISK is involved in rodents only. The different effects of humoral factors of animal or human plasma in rodent’s hearts may be due to the inter-species transfer differences. In addition, the signal transduction may be different in healthy or diseased subjects, in which confounding factors are present (See also below “Effects of common risk factors and confounders on STAT3 cardioprotection”).

Differently from the Hildebrandt et al. study [82], in which healthy volunteers were enrolled, in a recent clinical trial the effect of humoral factors of patients suffering acute coronary syndrome (ACS) has been evaluated [38]. In this study the role of STAT3 in inducing cardioprotection has been demonstrated. In particular, extracellular vesicles (EV) were collected from ACS patients without or with RIPreC before percutaneous coronary intervention (PCI) and tested i*n-vitro* and an *ex-vivo* IR models [38]. EV recovered from non-preconditioned patients induced protection against IRI, both in vitro and in ex vivo model. Pre-treatment of the isolated rat heart with the specific STAT3 inhibitor, Stattic, supports the idea that STAT3 is crucial for EV-mediated protection. Moreover, in these EV silencing of dual-specificity phosphatase 6, a member of the MAPK phosphatase family, prevented STAT3 phosphorylation and cardioprotection in the rat heart. EV collected from patients subjected to RIPreC were found non-protective. "Hyperconditioning" [227] has been proposed for explaining the loss of EV-RIPreC-mediated cardio-protection in these ACS patients, already suffering from ischemic events.

Another interesting study [97] suggested that the protection provided by RIPerC may be mediated not only by STAT3 pathway activation, which occurs only hours after the ischemia, but also by a more immediate neuronal response in pigs. Indeed, during the coronary occlusion the authors observed an attenuated ST-segment elevation in the RIPerC group, which correlated with reduced IS and was related to neuronal stimuli rather than circulating factors. In addition, it was found that splenectomy abolished RIPreC cardioprotection and the RIPreC-induced STAT3 phosphorylation in pigs and in rats [133]. Besides, in rats only, the vagal nerve seems to have a mandatory role in the transduction of RIPerC cardioprotection.

Despite the majority of the preclinical studies confirming a causal role of STAT3 in the protective mechanism of RIC, other players may intervene and species-specific differences may have a relevant influence too. Indeed, clinical evidence demonstrated the involvement of STAT5 in humans. In patients undergoing coronary artery bypass grafting (CABG) surgery, RIPreC was able to lower the troponin I levels and protect the myocardium, but STAT5 was the only STAT protein whose phosphorylation was significantly increased after applying RIPreC [56, 80]. Accordingly, in STAT5 KO mice, it has been reported that the cardioprotective effects against myocardial IRI by RiPreC are abrogated [30]. In particular, in these hearts, STAT5 was found to be crucial for RIPreC-mediated IS reduction through the PI3K/AKT survival pathway and the anti-apoptotic cascade, thereby confirming the importance of RISK in rodents. Conversely, STAT3 was found activated/phosphorylated in STAT5 KO mice and was not further phosphorylated by RIPreC, revealing that when STAT5 is deleted, STAT3 is phosphorylated, but this activation is not sufficient to reprogram RIPreC cardioprotection against IRI. Yet, in isolated mouse hearts with cardiomyocyte STAT3 deletion, STAT5 significantly increased following ischemic challenging [59]. However, in this study, the authors analyzed the cardiac function only and found that the STAT5 activation by IPrec in this STAT3 KO model is insufficient to rescue the cardiac function. From these two studies [30, 59] we can speculate that STAT3 is more implicated in cardiac function, whereas STAT5 is fundamental against IRI in rodents.

Clinical trials demonstrated the effectiveness of RIPreC in attenuating cardiac IRI and improving the short-term prognosis. In the work of Wu et al. [230], the induction of RIPreC in children with a severe congenital cardiac disease (tetralogy of Fallot) prior to cardiopulmonary bypass surgery, was able to reduce the length of intensive care unit stay, as well as postoperative Troponin I and Creatinine Kinase-MB levels. Interestingly, in the RIPreC group, they observed higher expression of cardiac Hif-1a, p-Akt, p-STAT3 in Y705, p-STAT5, p-NOS and less mitochondrial swelling compared to the control group.

In other human studies, the involvement of STATs was questioned. For instance, in patients with coronary artery disease who underwent CABG, RIPreC applied before surgery was protective, as demonstrated by the lower Troponin I level [99]. Mitochondria isolated immediately after RIPreC showed increased ADP-stimulated complex I respiration, ATP production and Ca^2+^ retention capacity, as well as reduced ROS production; the contractile function of atrial trabeculae dissected before surgery and subjected to HR protocol, was significantly improved by a RIPreC maneuver. Despite this, the expression and phosphorylation of proteins including STAT3 were not different between the RIPreC group and the control group. However, in this study, protein phosphorylation was analyzed in human atrial trabeculae after in vitro HR at only 1-timepoint; therefore, it is possible that changes in protein phosphorylation were missed.

In summary, although a number of experimental studies relating to the parallels between STAT3 and STAT5 exist, to better clarify the role of these two proteins in human myocardial IRI more studies are needed. From the existing studies on humans [18, 56, 80, 230] and on animals [30, 59, 79], it appears that their roles in cardioprotection are largely species-specific. In some species/conditions STAT5 can replace STAT3 in a sort of Yin/Yang relationship, in others STAT5 appears as part of a completely different pathway.

Yet, some clinical studies [139, 152, 172] do not support the cardioprotective potential of RIPreC and this may be due to different factors, such as comorbidities of the patients as well as the choice of anesthetic during surgery (See also below “Effects of common risk factors and confounders on STAT3 cardioprotection”).

### STAT3 in hypothermic conditioning

Hypothermia is known to preserve cardiac function and prevent mitochondrial damage upon cardiac IRI [163]. It is currently used in clinics to improve survival in post cardiac arrest settings [161]. STAT3 seems to be involved in the mechanism of protection underlying the hypothermic conditioning [88]. Huang et al. [88] studied the mitochondria of rat hearts subjected to cardiac arrest and resuscitation with or without hypothermia (32 °C) and they observed less mitochondrial swelling and mPTPs opening in the hypothermic group and amelioration of complex I/III activity of the ETC. Interestingly, the amount of Y705 phosphorylation and STAT3 expression in mitochondria was increased in hypothermic rats. The causal involvement of STAT3 was confirmed in vitro by the use of STAT3 inhibitor, which resulted in the abrogation of hypothermia-induced protection in H9c2 cells. Due to the relevance of this approach in the clinic, more studies on the mechanisms are needed.

### STAT3 in pharmacological conditioning

Cardioprotection can be achieved thanks to administration of drugs and molecules before or after a coronary occlusion, that are, respectively, pharmacological pre- and post-conditioning, which can be induced either with exogenous or endogenous agents. Even low doses of TNFα, administered as preconditioning or postconditioning agent, confer cardioprotection by modulating ROS production and inactivating pro-apoptotic proteins, such as Bad [43, 116, 118]. Indeed, ROS may trigger TNFα-induced cardioprotection and their scavenging inhibits STAT3 phosphorylation [116, 147]. There is evidence that JAK-STAT3 activation may be obtained by several other factors, such as bradykinin, opioids, ethanolamine, melatonin, resveratrol, erythropoietin, cannabinoid agonists, insulin and prostaglandins (for reviews see [71, 114, 115]). In addition, a link between HDL and STAT3 has been described [64].

Recently, as shown in rat hearts subjected to LAD ligation, morphine preconditioning induced an increase in pY705 STAT3 and phosphorylated JAK2 and a reduction in IS [45]. Upon STAT3 inhibition with Stattic, these effects are lost, but using cyclosporine A, the protection is restored, suggesting that the positive effect of morphine relies on the inhibition of mPTPs that occurs downstream of STAT3 activation. Another study [62] about morphine conditioning on LAD-ligated rat hearts, highlights that morphine-induced pY705 STAT3 and phosphorylated JAK2 are fundamental in the crosstalk with RISK pathway, which in turn leads to the regulation of GSK-3β and confers cardioprotection. Indeed, Akt and GSK-3β phosphorylations are abrogated in presence of AG490, the inhibitor of JAK2/STAT3, and at the same time, STAT3 phosphorylation was abolished in the presence of the Akt inhibitor, wortmannin. The same crosstalk was observed by Shravah et al. [187] on H9c2 cells treated with propofol, an intravenous anesthetic exerting anti-apoptotic effects on cardiomyocytes. Propofol treatment induced phosphorylation of STAT3 in Y705 and S727; both of them were detected for up to 30 min, while at 6 h only Y705 phosphorylation was sustained. This finding correlates with the observation of nuclear translocation of STAT3 after 4 h of propofol treatment.

Evidence about STAT3 in mediating cardioprotection via the regulation of mitochondrial function is also provided by the work of Lemoine et al. [125]. They demonstrated that the administration of atorvastatin at early reperfusion prevents mPTPs opening and that this is dependent on STAT3 and TNF-alpha signaling in human atrial trabeculae model of H/R injury. In particular, in human atrial trabeculae exposed to H/R, atorvastatin ameliorated the contractile force in comparison with H/R control and increased Y705 STAT3 and JAK2 phosphorylation. Atorvastatin lost its cardioprotective effect in presence of TNFα and STAT3 inhibitors, while the addition of cyclosporine A (mPTPs opening inhibitor) restored the protection. This indicates an involvement of the SAFE pathway in the protection by atorvastatin and places the inhibition of mPTPs opening downstream of the activation of TNFα, JAK2, and STAT3.

Sodium–glucose cotransporter-2 inhibitors (SGLT2) are emerging as a class of anti-diabetic compounds exerting unexpected beneficial cardiovascular effects [6]. Among the SGLT2 inhibitors, different mechanisms of cardioprotection have been reported. For example, empagliflozin reduces IS by increasing STAT3 phosphorylation at early reperfusion in in vivo hearts of mouse fed weed western diet to induce diabetes [7]. Moreover, in healthy mice, chronic, but not acute, empagliflozin administration reduces IS via the STAT3 pathway [160]. Yet, another SGLT2 compound, dapagliflozin, attenuates cardiac fibrosis by regulating the macrophage polarization via STAT3 signaling in infarcted rat hearts [123]. Distinct mechanisms of cardioprotection could be speculated among the various SGT2 inhibitors.

Many endogenous compounds used in pharmacological conditioning induce STAT3 signaling. For example, the obesity-related adipokine *leptin* protects against IRI in various tissues including gut, kidney, brain and heart in which leptin activates both RISK and SAFE pathways [193]. Actually, the cardiac leptin signaling has been demonstrated to be fundamental to the cardiac structure and function after MI, by attenuating hypertrophy and metalloproteinase activity, reducing cardiomyocytes apoptosis and inflammation, maintaining the left ventricle contractility and preventing left ventricular dilation [148, 149]. In particular, Mcgaffin et al. [149] performed LAD ligation on cardiomyocyte-specific leptin-deficient mice and they found reduced cardiac function associated with the loss of STAT3 signaling and STAT3-responsive gene transcription. Smith et al. [193], using isolated rat hearts treated with leptin at the beginning of reperfusion, found a decrease in IS as well as early (2,5 min) STAT3 Y705 phosphorylation and subsequent (15 min) Akt phosphorylation. The addition of AG490 caused the loss of leptin-induced protection but did not affect Akt levels. In vitro, they observed that leptin postconditioning reduced mPTPs opening; this effect was abrogated in presence of AG490, indicating that STAT3 may be involved in mitochondrial regulation.

Another hormone, ghrelin, has been tested in the context of cardioprotection. Ghrelin is a 28 amino acid peptide initially isolated from the stomach, whose acylation is essential for binding to growth hormone (GH)-secretagogues-receptor type 1a and its endocrine functions. Although lacking GHS-R binding, unacylated ghrelin shares several effects with acylated ghrelin. Ghrelin exerts different activities on the cardiovascular system, such as modulation of cardiac contractility, inhibition of cardiac and endothelial cells apoptosis and improvement of left ventricular function in IR heart [168]. Treatment of rats with ghrelin for 3 weeks, after induction of MI, improved LV contraction, decreased expression of pro-apoptotic proteins, determined myocardial suppressor of cytokine signaling 3 (SOCS3) expression (an inhibitor of JAK2/STAT3 signaling) and STAT1 phosphorylation, improved antioxidant activity and increased JAK2 phosphorylation and STAT3 phosphorylation in both Y705 and S727 in a rat model of MI with LAD ligation [47]. According to these results, the protective effect of ghrelin may be mediated by the inhibition of JAK1/STAT1 signaling, which leads to pro-apoptotic effects, and by the inhibition of SOCS3. Using cardiac-specific SOCS3 KO mice, it has been shown that SOCS3 is key factor that exacerbates the myocardial IRI development [155].

In ex vivo (isolated rat hearts) model and in vitro (isolated mouse cardiomyocytes) model of IR, Fuglesteg et al. [53] demonstrated that insulin induces cardioprotection at reperfusion, by activating STAT3. Again, the inhibition of STAT3 through AG490, negatively affected the activation of Akt, suggesting a crosstalk between these two mediators.

We mentioned above the importance of S1P in STAT3 activation. Actually, S1P is a proinflammatory mediator of allergic diseases and exerts its functions through five specific cell surface receptors (S1PR1-5) [105, 141, 216]. However, S1P is also a potent protectant against IRI [179, 214]. The S1P cardioprotection is mediated by interaction with types 1, 2 and/or 3 S1PRs leading to the activation of RISK and/or SAFE pathways [37, 150]. Specifically, S1P treatment at early reperfusion protected isolated mice hearts subjected to IR by activating both RISK and SAFE pathways [197]. In this model, the decrease in IS was abolished both with the use of AG490 and STAT3^−/−^ mice. Interestingly, they found a peculiar kinetic of STAT3 S727 phosphorylation in the different cell compartments: an increase in phosphorylation was found both in the nucleus and in the mitochondrial fraction at 7 min post-reperfusion, while it persisted at 15 min post-reperfusion only in the nucleus: STAT3 translocation to mitochondria may be related to its cardioprotective effect.

In conclusion, exogenous and endogenous compounds may induce pharmacological conditioning involving either RISK and/or SAFE pathways. Undoubtedly, further research is needed to delineate the main mechanisms involved in cardioprotection induced by these agents. Furthermore, the elucidation of the possible protective mechanisms of these factors on myocardium with or without comorbidities could increase the probability of success in terms of translating cardioprotection in the clinical setting for a real benefit of the patient. Since STAT3 is involved in a wide range of patho-physiological cardiac conditions, pharmacological activation or inhibition of STAT3 may be represent a therapeutic strategy to treat them.

## Some more considerations on mitochondrial STAT3 in IRI and cardioprotection

As seen above, STAT3 is a major member of the well-known cardioprotective SAFE pathway that leads to the activation of several pro-survival signals contributing to protect cells against IRI. There is a number of studies [23, 53, 83, 147, 157, 194] evidencing the essential role of STAT3 in cardioprotection against IRI, also through the use of STAT3 KO and pharmacological STAT3 inhibition. The KO of STAT3 and the inhibitors AG490 and Stattic are commonly used techniques to study the role of STAT3; these methods block all the functions of STAT3, without discriminating between transcriptional and mitochondrial function. Several studies showed that STAT3 is phosphorylated and activated in different timepoints of IR episode, but only few works have been done to distinguish and characterize the S727 and Y705 phosphorylation and their effects on STAT3 localization and function. A possible reason for this lack of insight can be due to the complexity of the model required to study separately STAT3 as a transcription factor and STAT3 as a mitochondrial effector, since no well-defined mechanism to distinguish them has been found yet. The different sites of phosphorylation, S727, and Y705, may represent a way to identify different activities of STAT3, but more studies should be done to verify if phosphorylation on S727 is specifically and univocally required for the mitochondrial action. Until now the S727 site seems to be important for some of the mitochondrial functions of STAT3, but it cannot be considered yet a specific marker to define mitochondrial STAT3. One interesting method that has been adopted by some research groups that wanted to focus only on mitochondrial STAT3 is the creation of MLS-STAT3, which is obtained by fusing the sequence of STAT3 with the mitochondrial targeting sequence of the subunit VIII of cytochrome c. The main disadvantage of this technique is that it forces the expression of a non-physiological mitochondria-targeted STAT3, and as such it does not allow to study of what happens physiologically in myocardium. Heusch et al. [79] observed that the reduction in IS caused by IPostC in pig hearts was accompanied by a better mitochondrial function, with preservation of complex I and better Ca^2+^ retention capacity; interestingly, in mitochondria from post conditioned pig heart they found an increased phosphorylation of mitochondrial STAT3 at the Y705 site. Among the few researchers that investigated the role of mitochondrial STAT3 in the IRI model, Szczepanek et al. [205] used transgenic MLS-STAT3 mice; in MLS-STAT3 hearts they found a better response to IR in term of IS and contractile recovery, as well as reduction of ROS after reperfusion, in comparison with WT hearts [204, 206]. The same group studied how mitochondrial function in particular complex I respiration, was improved by STAT3 overexpression in mitochondria, suggesting that the protective mechanism mediated by STAT3 is independent of its activity as a transcription factor. Wang et al. [220] have demonstrated that the zinc transporter Zip2 regulates mitochondrial respiration via STAT3 phosphorylation during IR. In particular, in Zip2^−/−^ mice undergoing an in vivo LAD ligation, mitochondrial respiratory rates and oxidative phosphorylation are decreased together to the levels of S727-phosphorylated STAT3. The restoration of mitochondrial function occurred only upon the reintroduction of STAT3 expression, while STAT3 dominant negative mutant (STAT3 S727A) inhibited mitochondrial respiratory function, suggesting the importance of the S727 site for mitochondrial regulation.

In cardiomyocyte STAT3 KO mice, Hilfiker-Kleiner et al. [83] observed increased mRNA levels of the pro-autophagy/pro-apoptotic protein BNIP3 24 h after reperfusion, while mRNA levels of the prosurvival gene HSP70 were decreased. They described greater apoptosis and IS with impaired fractional shortening after 7 days. However, no changes were observed in VEGF and in the anti-oxidant protein SOD2.

Yet, STAT3 signaling, other than regulating many responses in cardiomyocytes, is also active in the endothelial cells, where it contributes to anti-inflammatory and anti-apoptotic response [251], angiotensin II-induced fibrosis [86], oxidative stress modulation [7], and vasodilation [224]. According to this, endothelial STAT3 may have an important role in the protection versus IRI; for example, in mouse model of IR, the ablation of endothelial STAT3 led to a decrease of cardiac function supported by increased inflammation and worsening of capillary integrity [221]. Other authors demonstrated that in hypoxic human coronary artery endothelial cells, the treatment with statin rosuvastatin improved cell survival through the JAK/STAT3 pathway [218].

## Effects of common risk factors and confounders on STAT3 cardioprotection

Obviously, in AMI patients, it is possible to apply treatment only after the ischemia at early reperfusion, since the ischemic event cannot be predicted. As seen, pharmacological postconditioning at the early reperfusion phase promotes pro-survival and antioxidant mechanisms in the damaged cells and the treatment at late reperfusion appears to be less efficient [58]. Therefore, the studies about pharmacological postconditioning are the most relevant to clinical reality. As seen, STAT3 seems to have a protective role in various models of cardiac IR, even if different results about the timing and site of activation are present. Nevertheless, the results obtained in pre-clinical studies often do not correspond to the findings in clinical studies, suggesting that the species-specific differences in the responsiveness to treatment are strongly relevant. Yet, multiple factors including comorbidities and/or co-medications may influence the molecular environment in cardiac and non-cardiac tissues, determining the outcome in humans in the responses to RIC maneuvers [50, 98].

A known confounder of cardioprotection by RIC is the anesthesia. In this respect, STAT5 function may be relevant. As mentioned, it has been proposed that the SAFE pathway plays a more important role in larger mammals, where STAT5 is also important, making it particularly interesting from a translational viewpoint [70, 79, 189, 190]. Indeed, STAT5 phosphorylation is not only associated with, but causally involved in cardioprotection as suggested by the clinical study of Kottenberg et al. [101]. In this study propofol interfered with STAT5 activation and consequently with RIPreC-mediated protection, suggesting an association between a lack of RIPreC effect and the lack of STAT5 activation. Nevertheless, RIC decreased troponin release and increased STAT5 phosphorylation in patients undergoing cardiac surgery under isoflurane [80]. Augmented STAT5 phosphorylation in the left ventricle was also observed during cardioprotection under sevoflurane anesthesia [230]. The difference may be due to intravenous anesthesia (propofol) or volatile anesthesia, as suggested by the network meta‐analysis of randomized trials, which confirmed the interference of intravenous anesthesia with RIC in reducing post‐operative mortality [242].

Actually, a multitude of risk factors influences the onset and prognosis of ischemic heart disease, altering endogenous cardiac signaling and possibly interfering with cardioprotective strategies, including IPreC and IPostC [50, 98]. Since confounding factors such as age, sex, diabetes, and hypertension/hypertrophy may alter the benefits provided by the activation of the RISK and SAFE pathway, here we consider possible effects that these unfavorable factors may have on cardiac STAT3. Again, the majority of the evidence about confounder’s influence refers to STAT3 in the SAFE pathway, but a few studies can be linked specifically to the mitochondrial function of STAT3. Here, we consider some of these confounders and analyze the variations occurring on phosphorylation of S727 STAT3, the putative key step for STAT3 entry within mitochondria.

### Aging

#### Effects on the extent of IRI

Aging enhances the extent of IRI in unprotected myocardium due to age-induced defects in mitochondrial function as shown by Lesnefsky and Hoppel group [126, 127]. This may be related to STAT3 inhibition. Indeed, in aging rat heart lower phosphorylation of total STAT3 in comparison with younger animals has been observed [29]. Furthermore, Wegrzyn et al. [225] demonstrated that in mice STAT3 null hearts, the activities of complex I and II are decreased, leading to a reduction in the oxidative phosphorylation system (OXPHOS) efficacy.

#### Effects on cardioprotection

Aging is known to reduce the protection associated with ischemic conditioning, and a possible explanation may be the impairment of mitochondrial function and TNF-stimulated signaling, such as the SAFE pathway [18, 20, 126]. In aged rodents IPostC applied at reperfusion resulted in less efficiency in reducing IS in comparison with younger mice, and the increased IS was associated with reduced phosphorylation of S727 STAT3 [18]. In particular, in young mice (3 months) both 3 cycles of 10 s of ischemia/10 s of reperfusion (3 × 10 s) and 5 cycles of 5 s of ischemia/5 s of reperfusion (5 × 5 s) reduced IS, whereas in aged mice (> 13 months), only 5 × 5 s was effective in reducing the infarct area. In young mice, 3 × 10 s IPostC increased the phosphorylation of STAT3 in S727 at 10 min of reperfusion, whereas in aged mice hearts, phosphorylated STAT3 was reduced compared with young mice hearts. Other groups demonstrated that in the heart from old mice there is a lower expression of total STAT3. For example, Boengler et al. [20] reported a reduced STAT3 protein level in mitochondria isolated from cardiac tissue of aged mice compared to younger mice. Furthermore, in this study Boengler et al. [20] suggested the potential clinical relevance of STAT3 by highlighting that a reduced level of mitochondrial STAT3 in elderly hearts may be important for the loss of cardioprotection. Taken together, these studies highlighted the reduced STAT3 level in aged hearts with higher susceptibility to IR challenging and subsequent loss of cardioprotection.

### Diabetes

#### Effects on the extent of IRI

In addition to harmful consequences of diabetes on the cardiovascular system, such as cardiopathy and vasculopathy, diabetes increases specifically the risk to develop ischemic heart disease [34, 167]. Interestingly, diabetes seems to alter cardiac STAT3 signaling, lowering its activation at the S727 site in both in vivo and in vitro models [217], even if in some other cases, increased activation of canonical STAT3 was observed in diabetic animals [203]. Of note, STAT3 is known to protect the heart from oxidative stress ant it is able to preserve the myocardium in postpartum cardiomyopathy in female mice [84]. Indeed, a specific cardiac deletion of STAT3 in KO females leads to this disease [84]. The involvement of STAT3 in either the activation of diabetic or antidiabetic mechanisms in the development of diabetic and postpartum cardiomyopathy has been summarized in a recent review [66]. Despite not all evidence being coherent, diabetes contributes to worsening IRI, most likely through induction of oxidative stress and consequent loss of cardioprotective strategies efficacy [165]. For example, reduced STAT3 S727 phosphorylation in diabetic rats has been linked to increased susceptibility to IRI, resulting in increased IS [131, 217].

#### Effects on cardioprotection

Lei et al. [124] demonstrated that pharmacological preconditioning with Remifentanil in rats increased phosphorylation of STAT3 at Y705 and reduced IS. These protective effects were abrogated in diabetic rats, in which STAT3 failed to be phosphorylated both at Y705 and S727 [124]. The study of Liu et al. [136] investigated the influence of diabetes on RIPreC maneuver on rats. In particular, in diabetic rats subjected to liver RIPreC, IS and apoptosis level were reduced to the same extent as in non-diabetic rats. Interestingly, the cardioprotection of liver RIPreC in diabetic rats appeared to rely on GSK-3β/STAT5 signaling, since STAT5 activation and not STAT3 was detected in these hearts. Therefore, not all the evidence converges towards a single conclusion, but this can be due to the fact that diabetes can modulate the cardiac environment towards a less or more protective one in the different phases of its pathophysiology [167].

### Cardiac hypertrophy

Several studies highlighted that the inhibition of STAT3 induces downregulation of synthesis of collagen and subsequently regression of hypertrophy. Moreover, overexpression of STAT3 in murine cardiomyocytes leads to cardiac hypertrophy by increasing expression of hypertrophic genes, such as atrial natriuretic factor (ANF) [66, 106]. Regarding STAT3's role in mitochondrial regulation of cardiac hypertrophy, it protects mitochondria indirectly via upregulation of the anti-apoptotic proteins, such as Bcl2. In addition, STAT3 preserves mitochondrial integrity and inhibits ROS generation via its interaction with CypD and inhibition of the opening of the mPTPs [78, 87, 151]. Finally, Chen et al. [31] investigated whether angiotensin II-induced cardiomyocyte hypertrophy is affected by STAT3-mediated inhibition of cellular autophagy on H9c2. They reported that STAT3 modulates autophagy to balance the transcriptional hypertrophic response to angiotensin II stimulation.

Under stressful conditions, high levels of catecholamine are associated with several types of cardiac dysfunction, such as arrhythmias, coronary spasm, Ca^2+^ abnormalities in sarcolemma and sarcoplasmic reticulum, deficiency in mitochondrial energy production and myocardial cell damage, as well as cardiac hypertrophy [2, 90, 93]. Several factors have been proposed as promoters of hypertrophy [208]; among them, STAT3 was examined by Jeong et al. [93] in an in vitro model of catecholamine-induced hypertrophy. In this model they observed an impaired oxidative phosphorylation in mitochondria, as demonstrated by the downregulation of mitochondrial complexes II and III. In particular, they attributed a regulatory role to STAT3 phosphorylation at both Y705 and S727 sites in these hypertrophic H9c2 cells exposed to catecholamines. While the expression and activation of pY705-STAT3 in the nucleus were increased leading to the transactivation of hypertrophic responsible genes, such as ANF and c-fos, the pS727-STAT3 phosphorylation in mitochondria was attenuated. Moreover, the oxidative phosphorylation system of mitochondria was significantly downregulated, likely through the reduction of pS727-STAT3 activation.

Moreover, Stapel et al. [199] emphasize the critical implication of STAT3 in the sensitivity of β-adrenergic receptor (BAR) toxicity. They observed reprogrammed cardiomyocyte oxygen metabolism upon β-adrenergic stimulation in STAT3 KO mice, in particular an increased ADP/ATP ratio and lower O_2_ consumption.

In an effort to identify the potential involvement of STAT3 in β-adrenergic signaling Zhang et al. [246] studied BAR-mediated STAT3 activation, using conditional STAT3 KO (STAT3cKO) mice hearts. STAT3 should be involved in the regulation of BAR signaling via cAMP. In particular, in STAT3cKO hearts upon BAR stimulation cAMP level were downregulated and this was associated with lower left ventricular contractile and relaxation properties. This evidence was supported by the results obtained in the in vitro cardiomyocyte STAT3cKO model, where they found reduced anti-apoptotic proteins, indicating the activation of apoptotic processes.

Overall, these experimental studies suggest that a modulation of STAT3 plays a central role in the development of hypertrophy and may represent a new therapeutic strategy to treat cardiac hypertrophy. Whether these effects contribute to IRI exacerbation and loss of cardioprotection remains to be ascertained.

#### Effects on the extent of IRI

Several cardiovascular pathologies as well as hypertension can cause maladaptive hypertrophy of cardiac myocytes in response to an increased workload. When hypertrophy develops, IRI are exacerbated and cardioprotection is compromised; for Review see [165]. Moreover, Enomoto et al. [48] demonstrated that cardiac-specific ablation of the STAT3 gene exacerbated post-infarct cardiac remodeling. In particular, in STAT3 null mice, reduced capillary density was detected compared to control mice after myocardial infarction, suggesting that cardiac specific ablation of the STAT3 gene leads to severe hypertrophy without coordination with capillary growth. Overall, STAT3 downregulation by hypertrophic pathological conditions can exacerbates IRI, and STAT3 intrinsic activity is required for the prevention of maladaptive cardiac remodeling in the subacute phase of myocardial infarction.

#### Effects on cardioprotection

On the other hand, STAT3 downregulation by hypertrophic pathological conditions can also lead to the loss of efficacy of cardioprotective maneuvers, such as RIC [165]. In a clinical study, Sloth et al. [192] observed in 139 patients with STEMI and left ventricular hypertrophy that the RIPreC effect tended to be attenuated, even if no statistic differences were reached neither for patients with hypertrophy nor with hypertension. In the work of Song et al. [198], RIPreC was ineffective in inducing the activation of cardioprotective pathways (RISK and SAFE) when applied before aortic valve replacement in patients suffering from aortic stenosis, who are characterized by concentric left ventricular hypertrophy. CK-MB and troponin I levels were not affected by the RIPreC, as well as no changes in RISK pathway elements nor in STAT3 phosphorylation were detected. STAT5 phosphorylation was decreased in the RIPreC group, and caspase 3 was upregulated, suggesting that some cellular damage has occurred. These results lead to the conclusion that the presence of a hypertrophic phenotype influences the outcome of RIPreC maneuver, but the reason remains unclear. A possible explanation for lack of activation of the RISK pathway can be that in myocardium with chronic hypertrophy, a persistent activation of Akt exists [145], which might be linked to a feedback inhibition of upstream RISK pathway.

Overall, in several cardiac responses observed in hypertrophic hearts, such as stress adaptation, pathological remodeling, heart failure, IRI and cardioprotection modifications, STAT3 shows a fundamental function as a critical transcription factor and/or a modulator of mitochondrial function.

## Conclusion

Overall, in this review, we highlight the role of STAT3 as a protein with non-genomic functions. Initially, the research focused on the genomic functions of STAT3, as a transcription factor, but then it became clear that it has a fundamental role in the mitochondria and in the non-genomic network [e.g., 20, 210, 225], opening the horizon towards the understanding of the pro-survival functions of this protein.

In cardioprotection scenario, STAT3, by regulating mitochondrial Ca^2+^ and ROS homeostasis and preventing mPTPs opening, contributes to the recovery of mitochondrial function following IR episode, which is a fundamental prerequisite in the mechanism of efficient cardiac conditioning.

Although the most recent data suggest that optimal cardioprotection can be reached with the combination of additive or synergistic multitarget therapies [39], understanding how to preserve the ΔΨm and maintain the proton motive force by preserving the normal function of the F1F0-ATPase pump would undoubtedly result in an improvement in the response to IR. The available data on STAT3 in mitochondria and its influence on mitochondrial function represent a basis for further studies addressing the exact contribution of the role of mitochondrial proteins in cardioprotection.

Here we have discussed some recent studies that highlight the key role of STAT3 in cardioprotection, which, together with other regulatory (e.g., STAT5) and pathways, plays a central role also in humans and in experimental models relevant for translation from bench research to clinic [e.g., 79, 80, 98, 100, 101, 191].

We hope that the results reported in this review will encourage more and more researchers to deepen their studies and clarify the remaining doubts about STAT3 pro-survival role in the cardiovascular field. In particular, the function exerted on the ETC to maintain its efficiency and prevent ROS formation represents an excellent starting point to justify the interest in understanding the mechanisms underlying this role of STAT3 within mitochondria.
